# Exploring Multi-Anion Chemistry in Yttrium Oxyhydrides: Solid-State NMR Studies and DFT Calculations

**DOI:** 10.1021/acs.jpcc.3c02680

**Published:** 2023-07-17

**Authors:** Shrestha Banerjee, Diana Chaykina, Rens Stigter, Giorgio Colombi, Stephan W. H. Eijt, Bernard Dam, Gilles A. de Wijs, Arno P. M. Kentgens

**Affiliations:** † Institute for Molecules and Materials, Radboud University, Heyendaalseweg 135, NL-6525 AJ Nijmegen, The Netherlands; ‡ Materials for Energy Conversion and Storage, Department of Chemical Engineering, Delft University of Technology, Van der Maasweg 9, NL-2629 HZ Delft, The Netherlands; § Fundamental Aspects of Materials and Energy, Department of Radiation Science and Technology, Faculty of Applied Sciences, Delft University of Technology, Mekelweg 15, NL-2629 JB Delft, The Netherlands

## Abstract

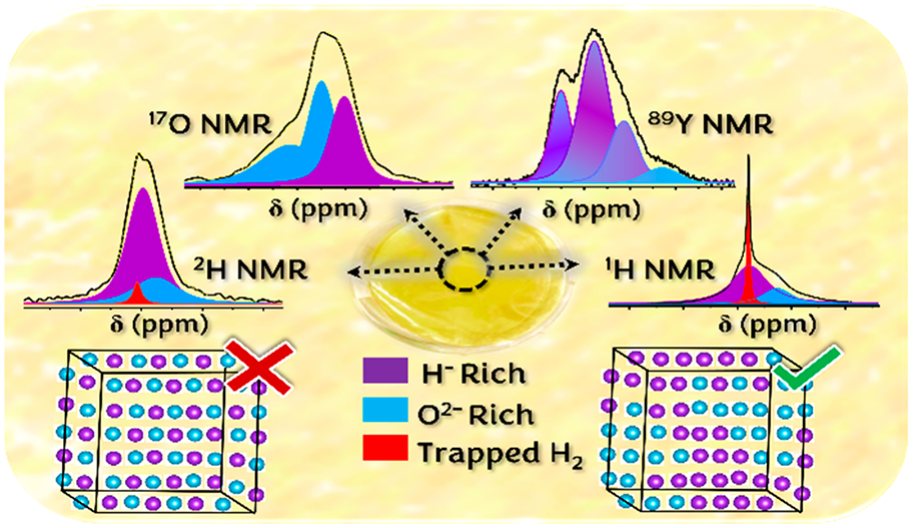

Rare earth oxyhydrides REO_*x*_H_(3–2*x*)_, with RE = Y, Sc, or Gd and a cationic FCC lattice, are reversibly photochromic in nature. It is known that structural details and anion (O^2–^:H^–^) composition dictate the efficiency of the photochromic behavior. The mechanism behind the photochromism is, however, not yet understood. In this study, we use ^1^H, ^2^H, ^17^O, and ^89^Y solid-state NMR spectroscopy and density functional theory (DFT) calculations to study the various yttrium, hydrogen, and oxygen local environments, anion oxidation states, and hydride ion dynamics. DFT models of YO_*x*_H_(3–2*x*)_ with both anion-ordered and anion-disordered sublattices are constructed for a range of compositions and show a good correlation with the experimental NMR parameters. Two-dimensional ^17^O–^1^H and ^89^Y–^1^H NMR correlation experiments reveal heterogeneities in the samples, which appear to consist of hydride-rich (*x* ≈ 0.25) and hydride-poor domains (*x* ≈ 1) rather than a single composition with homogeneous anion mixing. The compositional variation (as indicated by the different *x* values in YO_*x*_H_(3–2*x*)_) is determined by comparing static ^1^H NMR line widths with calculated ^1^H–^1^H dipolar couplings of yttrium oxyhydride models. The 1D ^17^O MAS spectrum demonstrates the presence of a small percentage of hydroxide (OH^–^) ions. DFT modeling indicates a reaction between the protons of hydroxides and hydrides to form molecular hydrogen (H^+^ + H^–^ → H_2_). ^1^H MAS NMR indicates the presence of a mobile component that, based on this finding, is attributed to trapped molecular H_2_ in the lattice.

## Introduction

Rare-earth oxyhydrides are an emerging class of multianion compounds that show a gamut of interesting optical and magnetic properties.^1−6^ The heteroanionic sublattice, consisting of oxide (O^2–^) and hydride (H^–^) ions, and specifically, the ionic ordering on this lattice control their properties.^4^ The anion arrangement has a significant influence on the hydride ion dynamics as well.^7^ Metal hydrides are well-known for their hydrogen storage properties,^8,9^ whereas rare earth oxyhydrides are utilized as a photocatalyst for ammonia formation^2,6,10^ and as H^–^ conductors.^5,11^ A recent study on cubic lanthanum-based oxyhydrides attributes this conductivity to the soft and polarizable nature of hydrides that facilitates ionic mobility.^11^ The low ionic mass, ample availability, and high polarizability thus make the hydride ion a very versatile anion.

The Sc, Y, and Gd oxyhydrides show color-neutral, reversible photochromism,^12^ the mechanism of which is still not well-known. The photochromism is induced by the photoexcitation of electrons of these semiconductors and is characterized by an unusually wide optical range.^13^ They are synthesized as thin films that are up to 1 μm thick. On exposure to UV/visible light, these materials change from a yellowish, translucent state to a dark, opaque state. They exhibit a considerable drop in optical transmission after UV illumination, as was first noted by Mongstad et al.^14^ The empirical formula for these oxyhydrides follows a specific compositional trend that is in between the trihydrides and oxides with the formula REO_*x*_H_(3–2*x*)_^13^ (assuming a full oxidation of the RE and oxygen and hydrogen to be in the 2– and 1– oxidation state, respectively). They are distinctly different from the hydroxides in terms of band gap, lattice structure, and photochromic properties.^15^ These materials are semiconductors, as is confirmed from optical transmission studies (Figure S1) whose band gap can be tuned by varying the O^2–^:H^–^ ratio during deposition.^16^

**Figure 1:**
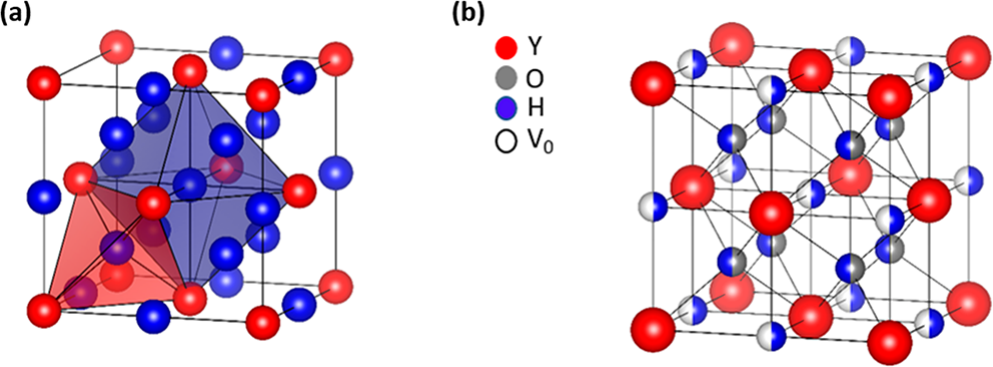
(a) Tetrahedral (red polygon) and octahedral (blue polygon) sites of the anion sublattice in an FCC lattice of a rare earth (here shown is the structure of FCC YH_3_) metal. (b) Yttrium oxyhydride FCC lattice. Oxygen atoms occupying only tetrahedral sites, hydrogen atoms occupying the remaining tetrahedral sites and octahedral sites further, and a few octahedral vacancies remaining.

The REO_*x*_H_(3–2*x*)_ oxyhydrides have a FCC cation lattice^17^ with the XRD patterns best matching the *Fm*3*m* space group of REH_2_.^16,18,19^ The anions occupy the tetrahedral and octahedral sites in the lattice (Figure 1), with a preference for the tetrahedral site, mainly because of the favorable lattice energy as compared to the octahedral sites.^18^ An EXAFS study has shown that oxide ions have a stronger preference for the tetrahedral sites than hydride ions and, hence, substitute the tetrahedral hydrides during air oxidation (Figure 1b).^18^ This is a consequence of a stronger ionic bond of the cation with the oxide, which is further rationalized by calculating the Madelung energy.^18^ The precise arrangement of the anions in the lattice remains, however, unknown. The photochromic efficiency is observed to change with the O^2–^:H^–^ ratio. The soft and polarizable nature of the hydride ion is in stark contrast with the hard and highly electronegative nature of the oxide ion. It has been observed that with such a contrasting nature of the anions, the local arrangement of the anion-sublattice plays a major role in establishing the properties of the material.^20−22^ Other relevant structural characteristics are coordination defects and the distribution of vacancies. Hence, understanding the local arrangement and dynamics of the anions in the rare earth oxyhydride lattice is important to comprehend the underlying mechanism of the photochromic effect.

In this work, we use ^1^H, ^2^H, ^17^O, and ^89^Y solid-state NMR and density functional theory (DFT) calculations to investigate yttrium oxyhydride thin film structures because NMR spectroscopy is particularly sensitive to the local structural environments of the nuclei. Solid-state NMR studies on various hydrogen storage materials, including binary, tertiary, and complex metal hydrides, have provided information about the local structure, arrangement, and dynamics of the hydrides.^8,23−25^ In this work, the different cation and anion environments, their oxidation states, the anion sublattice arrangement, and hydride ion dynamics are investigated. Quantitative ^1^H NMR studies provide information on the O^2–^:H^–^ ratio on the anion sublattice, which can be useful for correlating the anion composition to the photochromic properties. The NMR findings are further compared with DFT calculations of model yttrium oxyhydride structures, which are constructed for various O^2–^:H^–^ compositions (ranging from *x* = 0.25 to 1.25), including both ordered and disordered anionic arrangements. Our previous NMR study of thin film oxyhydrides^26^ indicated the presence of a very mobile hydride component that disappeared upon UV irradiation. More in-depth studies are needed, however, to gain insight into the structural effects leading to the photochromic behavior of these materials. In this study we perform a detailed computational and experimental investigation of these materials in a transparent (as-prepared) state.

## Experimental Section

### Materials and Synthesis

Yttrium oxyhydride powders were made by first depositing a thin “delamination” layer of Au (∼10 nm) onto a 3 in. glass wafer by magnetron sputtering of a gold target at 25 W and under a flow of Ar (20 sccm) at a deposition pressure of 0.3 Pa. Next, a roughly 1 μm thick hydride was deposited by reactive magnetron sputtering of a Y target (MaTecK, 99.9%) in an atmosphere of Ar/H_2_ at a flow ratio of 7:1. The deposition pressure was kept at 0.5 Pa. The input power was 100 W.^19^ The as-deposited film is YH_1.9+*x*_, but upon air exposure, the film oxidizes to the oxyhydride phase. As this happens, due to presence of the Au layer, the film delaminates as flakes and can be mechanically scraped from the glass wafer and studied in the NMR as powders.

Thin films of yttrium oxyhydrides were sputtered onto flexible FEP foils (3 in. diameter) by reactive magnetron sputtering of a Y target (MaTecK, 99.9%) and an input power of 100 W. Both YH*_x_*O*_y_* powders (with the gold flakes) and thin films (with FEP foils) deposited at 0.5 Pa deposition pressure were investigated in this study. The ^1^H spectra for these two kinds of samples were measured and compared to verify their compositional similarities which showed good agreement. The aforementioned samples will be referred to as the powder samples and the thin film samples, respectively, in the rest of the text.

For both hydrogenated and deuterated films, the Y metal target was sputtered in the presence of Ar and either H_2_ or D_2_ (99.9 at. %) gas with a flow ratio of 7:1, and a controlled deposition pressure (0.5 Pa). While some hydrogenated films were oxidized in air, others were oxidized by either dry O_2_ or isotopically enriched ^17^O_2_ (99.9 atom %). To achieve this, the sample was moved from the vacuum chamber to an oxidation cell in the glovebox without exposure to air. After sealing in the cell, >20 mbar of the appropriate gas was introduced, and the sample was left to oxidize for several hours. Yttrium dihydride was sputtered as a reference material in the same manner as the oxyhydrides, except with an input power of 200 W during reactive sputtering, and a deposition pressure of 0.3 Pa. It has been shown that at this pressure, the as-deposited film is too dense to allow oxidation to the oxyhydride phase.^16,18^ The H_2_ (Ar) deposition pressure determines the concentration of the incorporated O_2_ in the lattice, with higher deposition pressures allowing more oxygen to be absorbed by the YH_*x*_. Thus, the samples deposited at higher H_2_ (Ar) deposition pressures are expected to have a more oxide-rich overall composition. However, the deposition pressure was kept constant at 0.5 Pa for this study.

The samples were stored in an inert chamber under a continuous nitrogen flow to avoid further contamination with O_2_ or H_2_O. Two consecutive sputtering sessions were employed to create enough material to fill the 1.6 mm MAS NMR rotors with YH_*x*_O_*y*_ powder samples. YH_*x*_O_*y*_ powder samples, containing a metallic gold layer, were mixed with KBr, in a ratio of 2:5 (KBr:YH_*x*_O_*y*_), to avoid unstable spinning due to Eddy currents in the gold. The thin-film samples were rolled inside a glovebag and packed into rotors for the NMR experiments.

### Solid-State NMR

^1^H and ^89^Y Magic Angle Spinning (MAS) solid-state NMR spectra were acquired on a Varian VNMRS 850 spectrometer, operating at a magnetic field strength of 19.96 T, corresponding to a ^1^H Larmor frequency of 849.71 MHz and a ^89^Y frequency of 41.64 MHz. A 1.6 mm HXY triple channel Varian probe was used for ^1^H MAS and ^89^Y CP-MAS^27^ experiments. Empty rotor spectra were acquired to subtract proton background signals from the 1.6 mm Varian MAS probe and rotor. The probe was equipped with a low gamma tuning box to tune the ^89^Y channel. ^1^H single pulse excitation (SPE) and ^1^H spin echo spectra were acquired. 2D ^1^H–^1^H exchange spectroscopy (EXSY) was performed to study spin diffusion between ^1^H sites, using the same equipment as mentioned above. Nuclei with a low gyromagnetic ratio (Y, Rh, W), which have a poor sensitivity^28^ can be studied by combining MAS and proton detection.^29^ Inversely detected (via the ^1^H channel) heteronuclear correlation (HETCOR) experiments were performed to study the ^1^H–^89^Y interactions. In some HETCOR experiments, Lee Goldberg Cross-Polarization (LGCP)^30^ was performed to suppress ^1^H spin diffusion to selectively probe interactions between yttrium and hydrogen that are in close proximity.

Static ^1^H NMR experiments were performed on a 300 MHz Varian VNMRS spectrometer using a home-built, single-channel, static, proton-free probe with a RF coil diameter of 1.6 mm. The ^1^H Larmor frequency was 300.15 MHz. Adamantane (1.85 ppm) was used as the secondary reference for all of the ^1^H solid state NMR experiments. Yttrium chloride hexahydrate solution (4 M; 0 ppm) was prepared and mixed with 0.1 M MnCl_2_·4H_2_O for referencing the yttrium NMR spectra. The addition of MnCl_2_·4H_2_O to YCl_3_·6H_2_O helped to reduce the extremely long T_1_ values of the yttrium nucleus and optimize the acquisition time frame.

^2^H single pulse MAS (10 kHz) and variable temperature measurements were carried out only for the yttrium oxyhydride thin films on a 19.96 T (850 MHz) magnet. A 4 mm triple resonance Varian HXY MAS probe-head was employed. Deuterated water (4.5 ppm) was used as a secondary reference for these measurements.

Single-pulse ^17^O MAS^31,32^ spectra were measured for the yttrium oxyhydride thin film samples using a Varian VNMRS 600 MHz spectrometer (14.1 T) at a Larmor frequency of 81.35 MHz. A 3.2 mm HXY triple resonance Varian MAS probe-head was used for these measurements. A comparative field study was performed using a 400 MHz (9.4 T) Varian spectrum with a ^17^O Larmor frequency of 53.24 MHz. The probes used for these measurements were 3.2 mm HXY triple-channel Varian MAS probes. Spectra were referenced to the ^17^O resonance of water at 0 ppm. ^17^O–^1^H correlation experiments were performed on the 14.1 T magnet employing the PRESTO (phase-shifted recoupling effect with a smooth transfer of order) recoupling experiment. PRESTO is a symmetry-based pulse sequence utilizing R18^ν^_n_ type recoupling pulses on the ^1^H channel quadrupolar nucleus (^17^O *I* = 5/2).^33^^17^O-labeled l-tyrosine [S2 (Supporting Information (SI))] was used to optimize the experiments.^34−36^

All experiments were performed in a dry nitrogen atmosphere. The solid-state NMR spectra were processed and fitted using the ssNake software package.^37^ Detailed information about the acquisition of the NMR experiments can be found in S19 (SI).

### DFT Calculations

Electronic structure and chemical shielding calculations were carried out with the Vienna ab initio simulation package (VASP)^38,39^ using the projector-augmented wave (PAW)^40,41^ method with the Perdew–Becke–Ernzerhof (PBE)^42,43^ exchange-correlation functional. The gauge-including PAW (GIPAW)^44,45^ method was used for the shielding calculations.

The empty 4d orbitals of Y are typically placed at too low energies by semilocal DFT. This can lead to an overestimation of the covalency of the Y–O bond and, therefore, substantial errors in calculated shieldings. This error can be repaired with the DFT+U method.^46^ We checked the effect of a reasonable Hubbard U of 2.8 eV and observed only minor effects on the calculated chemical shifts [<3%, see S26 (SI)]. Hence, for our purposes, we do not need DFT+U and do not apply it here.

The Kohn–Sham orbitals were expanded in plane waves with a kinetic energy cutoff of 600 eV. The Brillouin zones were sampled with 8 × 8 × 8, 2 × 2 × 2, and 1 × 1 × 1 Γ-point centered k-point grids for structural optimization of the ordered structures, small cell, and large cell disordered structures, respectively (vide infra). Increasing the supercell volume results in a smaller Brillouin zone. Therefore, a larger cell requires fewer k-points to achieve the same k-point mesh density. A test was done for the YHO (*x* = 1 for YO_*x*_H_(3–2*x*)_) disordered structure with 6 × 6 × 6 k-points to ensure the convergence of the lattice and NMR parameters.

Standard VASP PAW potentials were used for yttrium, oxygen, and hydrogen (these have default cutoff energies of 203, 400, and 250 eV, respectively). The Y and O potentials have [Ar]3d^10^ and [He] frozen cores.

An yttrium FCC backbone with different heteroanionic ratios and arrangements was constructed. Previous studies have shown a clear preference of oxygen tetrahedral sites.^18^ Hence, we first placed all oxygen atoms in the tetrahedral sites and filled the remaining tetrahedral sites with hydrogen atoms. The remaining hydrogens occupy the octahedral sites.

Both ordered and disordered anion sublattice models were used. We introduced most of these in ref (50), where complete details can be found. Below we briefly summarize the structural models. Because here we use a different kinetic energy cutoff and Brillouin zone sampling than in ref (50), for consistency, we reoptimized the structural parameters, giving only very small differences.

The anion ordered structures were constructed to investigate the nature of ordering of heteroanions in a CaF_2_ type FCC unit cell (containing 4 Y atoms). The lattice constants obtained after cell optimization ranged from 5.23 to 5.34 Å depending on the composition, as the lattice constant increases with an increasing number of oxygen atoms in the structure. The formula YO_*x*_H_(3–2*x*)_ was utilized for constructing the unit cell, with varying the *x* values as 0.25, 0.5, 0.75, 1, and 1.25. Several space groups were tested for each composition to obtain different ordering schemes of the oxides and the hydrides. In the end, the lowest lattice energy structures [after structural relaxation; S3 (SI)] were selected.

The DFT calculations for the disordered anion lattice were divided into two groups. The first group consists of special quasi-random structures (SQS).^47^ These have a FCC lattice of yttrium containing 32 atoms, i.e., a supercell consisting of 2 × 2 × 2 conventional FCC cells and a lattice constant of 10.65 Å. The anions occupy the tetrahedral and octahedral sites and observe the constraints outlined above; i.e., all oxygens are on tetrahedral sites, all tetrahedral sites are occupied, and any remaining hydrogen is put at the octahedral positions that are not all filled. The total number of anions depends on *x*, which was varied as 0.25, 0.5, 0.75, 1, and 1.25. SQS structures are ordered structures that closely reproduce a perfectly random arrangement of the anion sublattice for the first few shells around given site and, hence, may comprise of relatively small supercells (details can be found in ref (50)). The second group consists of very large FCC supercells consisting of 4 × 4 × 4 conventional cells, containing 256 yttrium atoms, and a lattice constant of 21 Å. Here, the anions are put randomly on their sublattice while still observing the constraints outlined above. These supercells are introduced for the first time in this work and only for *x* = 0.25 and 1, which correspond to hydride-rich and hydride-poor compositions, respectively. These two compositions were best matched to the compositional variation obtained via NMR experiments (vide infra). In these cells, the disorder extends to larger distances than in the smaller SQS models. The large cells have NMR parameters in close correspondence with those of the smaller SQS cells [S20 (SI)], but offer improved statistical quality for, in particular, the Y coordinations.

The structures were relaxed using an electronic convergence threshold of 10^–6^ eV. The convergence criterion for structural optimization was 0.001 eV. First, the atomic positions, shape, and size of the YH_(3–2*x*)_O_*x*_ cells were relaxed. Subsequently, these well-converged structures were used for NMR shielding calculations.

The references used for the Y, O, and H nuclei are yttrium hydroxide (Y(OH)_3_), yttrium oxide (Y_2_O_3_), and adamantane, respectively. The calculated shieldings, σ_cal_, are converted to chemical shifts, δ_cal_, by using the following equation:
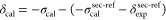
where σ_cal_^sec-ref^ is the calculated chemical shielding of the secondary reference and δ_exp_^sec-ref^ is the experimental chemical shift of the secondary reference (with respect to the primary reference, which is 0 in all cases). The δ_exp_^sec-ref^ values for Y (yttrium hydroxide (Y(OH)_3_)), O (yttrium oxide (Y_2_O_3_)), and H (adamantane) are obtained as 65, 356, and 1.85 ppm, respectively. The calculated NMR parameters were simulated as Gaussian peaks utilizing Matlab [S18 (SI)] and Fortran scripts.

The second moments were calculated using the Van Vleck equations^48,49^ and multiplied with √(8 ln(2)) to obtain the theoretical line widths for a rigid yttrium hydride (YH_2_ and YH_3_) and the yttrium oxyhydride lattices with varying *x* values (0.25, 0.5, 0.75, 1, 1.25). These values are compared to the static Gaussian line width of ^1^H for the oxyhydrides to assign a specific *x* value to each of the components of the deconvolution.

## Results and Discussion

### ^1^H and ^2^H MAS Solid State NMR and DFT Calculations

**Figure 2:**
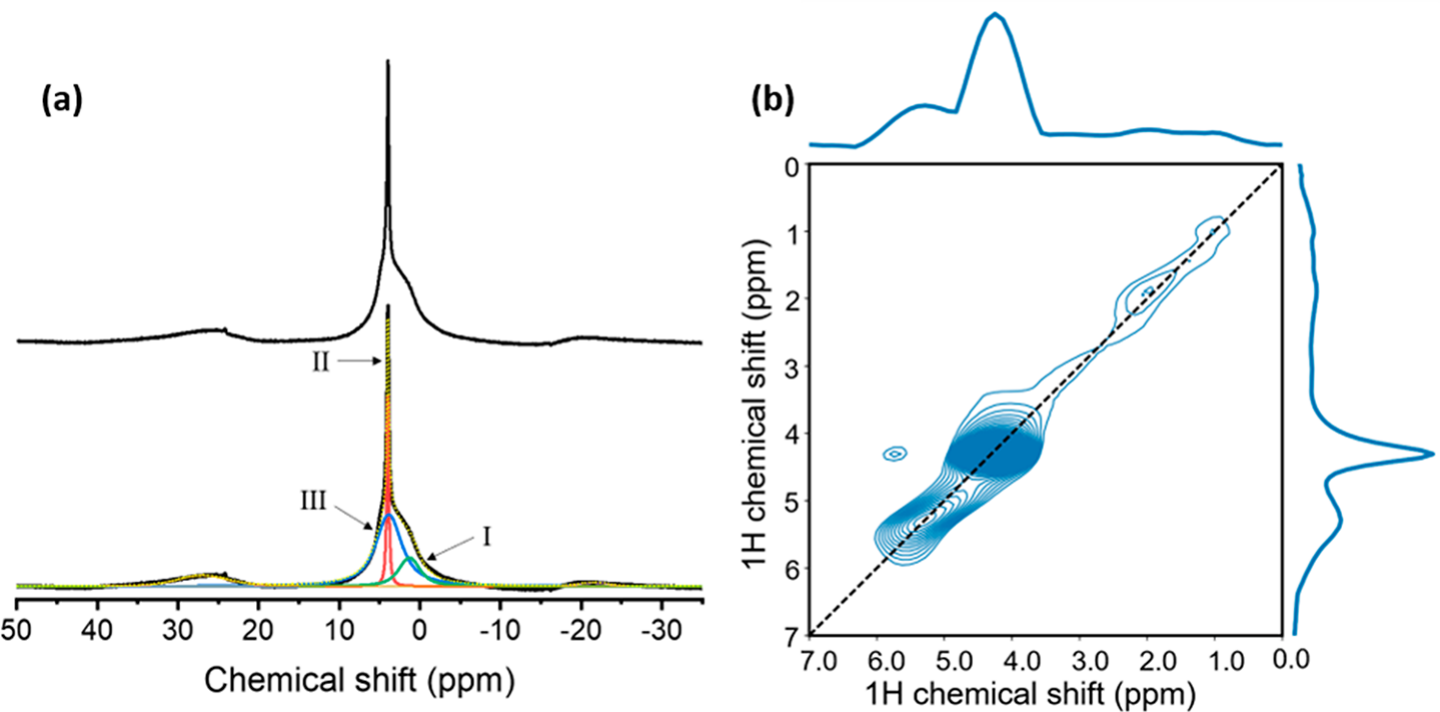
(a) ^1^H MAS spectrum of 0.5 Pa yttrium oxyhydride powder at 20 kHz MAS, 64 transients (top), and the deconvolution of this spectrum in three components labeled I, II, and III (bottom). (b) 2D ^1^H exchange spectroscopy (EXSY) of yttrium oxyhdride powder at 20 kHz MAS and 32 transients using a 4 ms mixing time. Both spectra were recorded on an 850 MHz spectrometer.

To understand the structure and composition of YH_*x*_O_*y*_, we first investigated the H-sites in YH_*x*_O_*y*_ powders (0.5 Pa). The ^1^H MAS spectrum in Figure 2a, can be deconvoluted into three components. These are centered at 2.8, 4.6, and 4.8 ppm (Table 1) and are denoted peaks I, II, and III, respectively. The resonance (II) at 4.6 ppm is very narrow, meaning that the dipolar interactions are extremely small. Therefore, this resonance is due to either very isolated protons, which is unlikely in these materials, or a highly mobile H species. Moreover, peak II has a very long spin–spin relaxation time (*T*_2_) compared to the broad peaks (S4), which indicates that this peak has a weak dipolar interaction with the neighboring spins. Consequently, peak II can be attributed to a highly mobile species for which the dipolar interactions to neigboring spins are averaged. The ^1^H chemical shift information is insufficient to assign this peak to an exact chemical species. However, subsequent ^17^O NMR spectroscopy and DFT modeling indicate that this resonance could be due to molecular hydrogen trapped in the structure (vide infra).

**Table 1:** Comparison of ^1^H and ^2^H MAS NMR Spectra of the YH_*x*_O_*y*_ and YD_*x*_O_*y*_ Thin Films^a^

nucleus	peak	position (ppm)	normalized integral	line width (kHz)	line width (ppm)
^1^H	I	2.8 (0.3)	0.21 (0.02)	3.5 (0.50)	4.05
	II	4.6 (0.1)	0.10 (0.01)	0.22 (0.05)	0.26
	III	4.8 (0.4)	0.69 (0.07)	3.2 (0.30)	3.8
^2^H	1	3.4 (0.4)	0.17 (0.02)	0.80 (0.10)	6.09
	2	5.1 (0.2)	0.11 (0.01)	0.07 (0.01)	0.56
	3	4.9 (0.4)	0.72 (0.07)	0.34 (0.08)	2.60

a The confidence intervals are given in parentheses.

**Figure 3:**
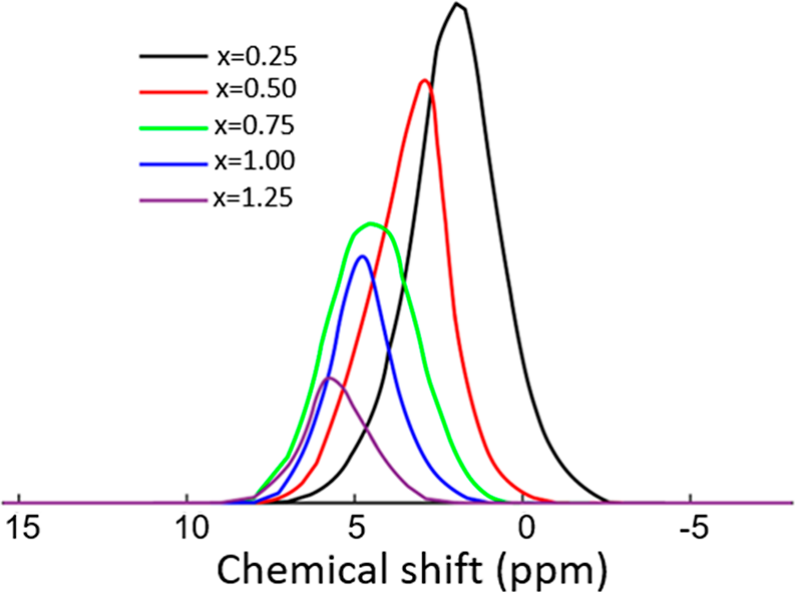
DFT calculated distribution of ^1^H chemical shift for model YO_*x*_H_(3–2*x*)_ structures with a disordered anion arrangement (or SQS structures) as a function of composition *x* (after lattice relaxation).

The lines at 2.8 ppm (I) and 4.8 ppm (III) have a substantial line width. Experiments as a function of external field strength [S5 (SI)] show that this line width is to a large extent determined by a distribution in chemical shift that could be the result of disorder in the structures. The two-dimensional exchange (EXSY) spectrum shown in Figure 2b confirms that there are, indeed, three distinct ^1^H environments. The contours are distributed along the diagonal, confirming a distribution in chemical shift for resonances I and III. The absence of cross peaks between the different resonances implies that the corresponding protons are spatially separated. Furthermore, the experimentally observed chemical shift distribution of the ^1^H peaks is replicated in the DFT modeling of disordered anion lattices (Figure 3). The anion ordered structures show, in turn, only a narrow distribution of chemical shifts for ^1^H [S8 (SI)] and the other nuclei (vide infra), which is not in line with our experimental results. Moreover, a recent computational study by Colombi et al. points out the preference for a disordered anion lattice over an ordered lattice, due to favorable energetics and hence stable lattice formation after structural relaxation.^50^ Therefore, we rule out the presence of ordered structures.

The ideal anion disordered sublattice is expected to contain two distinctly different hydrides at the tetrahedral and octahedral sites. However, DFT calculations on anion disordered models suggest that the hydrides are considerably displaced from their respective tetrahedral and octahedral positions after structural relaxation, thus attaining positions at intermediate locations between the ideal octahedral and tetrahedral lattice sites. The proximity of the octahedral and tetrahedral hydrides results in this displacement that is described in further detail in ref (50). As a result, the ^1^H chemical shifts of the originally tetrahedral and octahedral hydrides strongly overlap after lattice relaxation [S6 (SI)]. Therefore, the observed ^1^H peaks in the spectrum cannot be assigned to separate tetrahedral and octahedral ^1^H sites. Previous NMR studies on metal hydrides point to a direct proportionality between ^1^H chemical shift and metal to hydride distance.^3^ Therefore, the distribution in ^1^H chemical shifts obtained for components I and III reflects the distortion of the disordered anion sublattice. Hence, each of the components I and III contain contributions from hydrides displaced from both tetrahedral and octahedral sites. This is reflected in the chemical shift calculations for different compositions represented in Figure 3.

**Figure 4:**
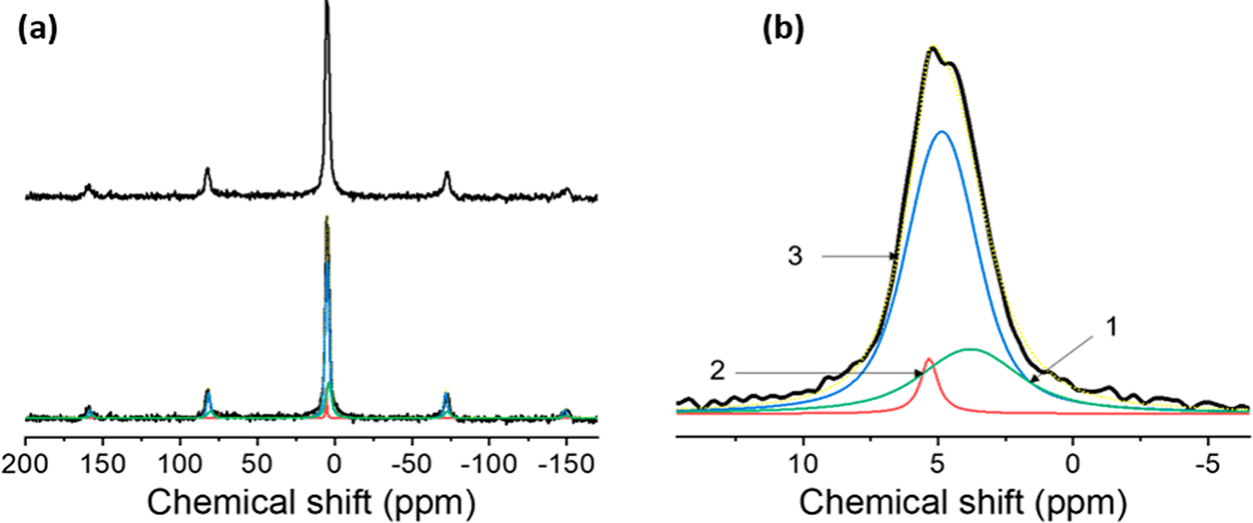
(a) (top) ^2^H MAS spectrum of yttrium oxyhydride thin film, (bottom) deconvoluted spectrum, and (b) ^2^H sites obtained after deconvolution (magnified deconvolution). The spectrum was acquired at 19.96 T magnet, employing a spinning frequency of 10 kHz and accumulating 1000 scans.

^2^H MAS NMR was employed to overcome the effect of residual ^1^H dipolar couplings and to obtain well-resolved hydrogen chemical shifts^51−53^ as ^2^H NMR does not suffer from substantial dipolar broadening because of its low gyromagnetic ratio (compared to ^1^H). ^2^H NMR studies of YD_*x*_ systems (1.98< *x* < 2.08) have shown enhanced hydride ion mobility with increasing temperature and exchange between tetrahedral and octahedral sites.^54^Figure 4a,b displays the ^2^H MAS NMR spectrum of a ^2^H isotope-labeled yttrium oxyhydride thin film. The deconvolution of the spectrum included the spinning sideband manifold reflecting the first-order quadrupolar interaction (Figure 4a). The spectrum corresponds to three overlapping resonances (Figure 4b) at positions closely corresponding to the ^1^H MAS spectrum (Table 1). The appearance of the narrow component (peak 2) in the ^2^H spectrum confirms the presence of a mobile component as a part of the structure. Component 2 has no detectable quadrupolar interaction that again hints at averaging of anisotropic (now quadrupolar) interactions due to mobility. The MAS line widths for both peaks in ppm are comparable (both ranging from 1 to 10 ppm), which confirms that they are dominated by a distribution in chemical shift.

To summarize, based on the line width of the ^1^H and ^2^H MAS NMR spectra, which originate from chemical shift distributions, we conclude that there is substantial disorder in the distribution of the anions in the lattice. This is corroborated by DFT calculations of anion-disordered oxyhydrides. The lattice relaxation of these model structures further increases the disorder by displacing the hydride ions from their ideal octahedral and tetrahedral positions. The 2D EXSY spectrum clearly shows that there are three ^1^H chemical environments that are not in close contact with each other. Except for peak II, peaks I and III are dominated by a distribution of chemical environments because of the disorder. The DFT calculations (Figure 3) indicate that each composition of the oxyhydrides displays a distinct chemical shift distribution, showing a deshielding trend as *x* (in YO_*x*_H_(3–2*x*)_) increases. These distributions overlap when comparing different compositions due to their large width. Therefore, the spatially separated ^1^H chemical environments observed experimentally could be due to a compositional variation in the anionic arrangement, i.e., each of the ^1^H peaks might correspond to a domain in the sample with a specific *x* value. These results exclude a uniform composition with a single *x*-value with random arrangement of the hydrides and oxides, in which case a single close to Gaussian distribution would be observed (Figure 3) and this is evidently far from our experimental findings. The cross-sectional SEM studies of a 270 nm yttrium oxyhydride thin film reported previously by Nafezarefi et al., revealed that the deposited film displayed a columnar morphology, which could result in a compositional variation.^84^

### Static ^1^H Solid-State NMR and DFT Calculations

**Figure 5:**
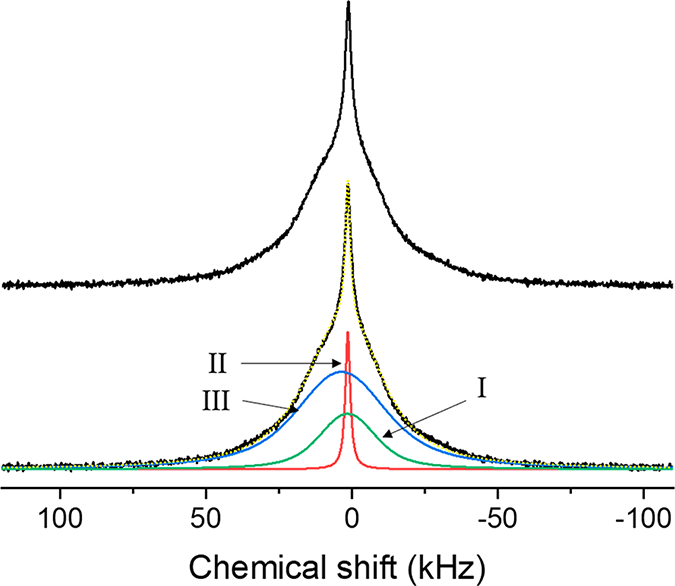
(top) ^1^H static spectrum of yttrium-oxyhydride thin film; (bottom) deconvoluted spectrum. Peaks I, II and III have line widths of 23.5 kHz, 2.9 kHz and 42.1 kHz, and integral ratios of 0.21, 0.10, 0.69 respectively. The spectrum was acquired at a 300 MHz NMR spectrometer using a static, proton background free 1.6 mm probe.

Static ^1^H measurements are employed to explicitly study the rigidity of the H anion lattice as a function of composition^26^ by correlating the experimental line widths to those predicted by DFT-based calculations. The ^1^H static spectrum for the yttrium-oxyhydride thin film in Figure 5 shows the presence of a narrow Gaussian component and two broad Gaussian components, as is shown in the deconvolution in Figure 5 (bottom). For the deconvolution, the integral ratios were restrained to those obtained from the ^1^H MAS spectrum (Table 1). The three Gaussian contributions are observed to have distinct line widths of 42.1, 23.5, and 2.9 kHz, which are associated with peaks III, I and II of the ^1^H spectrum (Figure 5) respectively. Below we will correlate this to the calculated second moments obtained from our DFT model yttrium-oxyhydride systems using the van Vleck equations for dipolar line widths^48,49^ (see Table 2). The mobile narrow fraction (peak II), which has been noted in the previous static ^1^H NMR experiments,^26^ accounts for nearly 10% of the total peak integral. Static line widths of peaks I and III in the absence of MAS, can be assumed to be predominantly determined by ^1^H–^1^H dipolar couplings, if the lattice is rigid. In that case, there is no effect of mobility that could partly average the dipolar interactions (and thus reduce the line widths).

**Table 2:** Calculated ^1^H Line Width of Rigid Lattices of Model Compounds (Yttrium Dihydride, Yttrium Trihydride, Yttrium Hydroxide) and Yttrium Oxyhydride (YO_*x*_H_(3–2*x*)_) Special Quasi Random Model Structures (*x* = 0.25, 0.50, 0.75, 1.00, 1.25) Using the Van Vleck Equations^a^

structure	space group	calcd line width (kHz)
YH_2_	*Fm*3*m* (ref ^78^)	31.6 (8c)
YH_3_ (HoD_3_ structure)	*P*3*c*1 (ref ^79^)	44.6 (12g), 41.9 (2a), 42.7 (4d)
Y(OH)_3_	*P*63/*m* (ref ^80^)	35.6
YO_0.25_H_2.5_		41.7
YO_0.50_H_2.0_		36.7
YO_0.75_H_1.5_		31.2
YO_1_H_1_		23.0
YO_1.25_H_0.5_		15.3

a Column 3 contains the line width of the specific ^1^H sites of model compounds (identified by Wyckoff positions in parentheses).

Comparing the DFT calculated ^2^H quadrupolar coupling constants C_Q_ [S16 (SI)] with experiments (Table 3), the ^2^H quadrupolar coupling constant data show no evidence of mobility for the two broad components, as the experimental values agree well with the calculations for static lattice models. This is further confirmed by low temperature ^2^H measurements [S7 (SI)] in which the line widths of the resonances remain independent of temperature. Therefore, the experimental static ^1^H line widths can be interpreted to be dominated by the ^1^H–^1^H dipolar interactions in a rigid lattice. The contribution from chemical shift distributions, as observed in ^1^H MAS spectra (vide supra), is minor compared to the size of the dipolar couplings. Table 2 shows the calculated ^1^H line widths of the model structures for a range of *x* values. We observe that the 42.1 kHz Gaussian peak closely matches the calculated line width for the model structure with *x* = 0.25, whereas the 23.5 kHz peak compares well to the *x* = 1 model structure, both having a disordered anion arrangement. Therefore, we attribute the two broad Gaussian resonances to a hydride-rich (*x* ∼ 0.25) and a hydride-poor (*x* ∼ 1) part of the anion sublattice. For the ^1^H MAS spectrum, this implies that the 4.8 ppm peak (III) corresponds to a hydride-rich domain and the 2.8 ppm peak (I) corresponds to a hydride poor domain. This is, however, contradictory to the trend that is observed for the calculated ^1^H chemical shift distribution (Figure 3), where the hydride-poor structures (*x* ≥ 1) show higher chemical shift values compared to the hydride-rich structures (*x* ≤ 1). More advanced modeling partially lifts this contradiction (vide infra). From a theoretical point of view, total energy calculations show a slight preference for segregation into a hydride-rich (*x* = 0.25) and hydride-poor (*x* = 1) composition compared to intermediate compositions (0.25 < *x* < 1 in YO_*x*_H_(3–2*x*)_), for the same average *x* [as discussed in S25 (SI)]. The intermediate compositions show a higher total energy for both yttrium and lanthanum oxyhydride systems. This is consistent with our experimental observations.

**Table 3:** Experimental Quadrupolar Parameters Obtained for ^2^H and ^17^O Measurements of YH*_x_*O*_y_* Thin Films Deposited at 0.5 Pa

nucleus	peak	position (ppm)	normalized integral	expt. ⟨|*C*_Q_|⟩ (MHz)
^2^H	1 (H^–^)	3.4 (0.4)	0.17 (0.02)	0.012 (0.002)
	2 (H_2_)	5.1 (0.2)	0.11 (0.01)	0.0002 (0)
	3 (H^–^)	4.9 (0.4)	0.72 (0.09)	0.015 (0.003)
^17^O	i (O^2–^)	355 (22)	0.27 (0.03)	0.67 (0.03)
	ii (O^2–^)	375 (29)	0.38 (0.05)	0.43 (0.04)
	iii (O^2–^)	402 (37)	0.30 (0.02)	0.98 (0.07)
	iv (OH−)	110 (12)	0.07 (0.01)	4.2 (0.5)

Quantitative ^1^H studies [S23 (SI)] were performed by employing a proton free probe to avoid background signals. Using glycine (C_2_H_5_NO_2_) as an intensity reference, *x* ∼ 0.48 is obtained for the average composition of the oxyhydride sample, based on the integrated intensities of weighed amounts of sample [S23 (SI)].

If we assume that the sample consists of regions with different compositions, we can estimate the fractions of these hydrogen-rich (*x* ∼ 0.25) and hydrogen-poor (*x* ∼ 1) domains based on their integrals in the ^1^H spectra. The ^1^H MAS spectrum gave relative integrals of 0.69 (*x* ∼ 0.25 region), 0.21 (*x* ∼ 1 region), and 0.1 (for trapped H_2_). Excluding the trapped H_2_, the relative amount of hydrogen-rich materials is 0.77/2.5= 0.31 versus 0.23/1 for the hydrogen-poor regions, where 2.5 and 1 correspond to the number of H in YO_*x*_H_(3–2*x*)_ for *x* = 0.25 and 1, respectively. The average composition based on this assumption can thus be calculated as , giving an average composition of *x* ∼ 0.55, which comes close to number obtained from the quantitative ^1^H NMR experiments. So we conclude that the sample consists of hydrogen-rich and hydrogen poor domains in a ratio of 3:2 and that its average composition is approximately YO_0.5_H_2_

### ^17^O Solid State NMR and DFT Calculations

**Figure 6:**
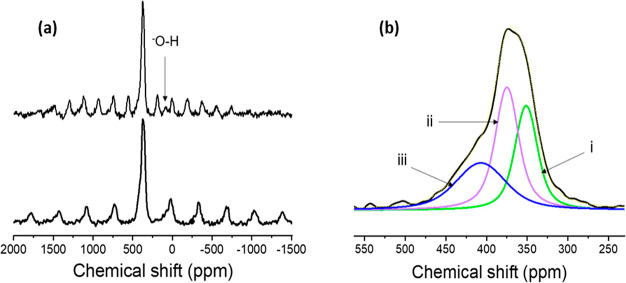
(a) ^17^O MAS spectrum of yttrium oxyhydride thin film at 15 kHz MAS acquired at 14.1 T (top) and 19 kHz MAS (bottom) acquired at 9.4 T. 4000 scans were accumulated per experiment. (b) Deconvoluted ^17^O spectrum obtained at 19 kHz spinning speed, zooming in on the central transition.

^17^O solid-state NMR has been extensively employed in recent years, specifically because of its wide chemical shift range and the high sensitivity of its quadrupolar coupling constant to the local symmetry.^55−59^ Here we use ^17^O NMR to obtain a deeper understanding of the structure of the anionic sublattice. The ^17^O NMR spectrum for the isotope labeled yttrium-oxyhydride thin film (Figure 6a) shows the central transition (CT, expanded in Figure 6b) and the spinning sideband manifold of the satellite transitions due to the quadrupolar interaction. The CT resonances cover a chemical shift range from 300 to 475 ppm, which is in the same range as the ^17^O spectra of yttrium oxide,^60^ yttrium stannate^61^ and yttrium titanate.^61^ The peaks can therefore be assigned to oxides in the anion lattice. The calculated ^17^O chemical shifts for anion disordered SQS model structures with oxygen in tetrahedral sites (S9) indicate a similar chemical shift range. To confirm the assignment of the oxide resonances to tetrahedral sites, a model structure containing oxide ions in octahedral sites was simulated (S10). The calculated ^17^O chemical shifts for these octahedral oxides are significantly lower (248 ppm) and hence are distinctly different from those of the tetrahedral sites. Moreover, it is observed in the DFT calculations and also noted in our previous theoretical study^50^ that, unlike the hydrides, the oxides are not displaced significantly from their tetrahedral sites after lattice relaxation and therefore resonate in a different chemical shift range than the octahedral oxides. The spectrum in Figure 6a is deconvoluted, including the spinning sideband manifold of the satellite transitions (full deconvolution shown in S15) and assuming a Czjzek distribution of the quadrupolar parameters. The resulting fit shows three Gaussian lines centered at 355, 375, and 402 ppm for the central transition (Figure 6b). All of the resonances are observed to have a relatively small quadrupolar coupling constant *C*_Q_ (Table 3). The small *C*_Q_ observed for the oxides hint at a rather symmetrical local environment, as expected for (near) tetrahedral coordinations. As a result, the second-order quadrupolar line broadening of the CT is negligible. This was verified by measuring at a different magnetic field strength (14.09 T) and comparing the line widths. The second order quadrupolar line broadening scales inversely with external magnetic field. In this case, however, the two spectra, as shown by the spectral overlay in S11, have nearly identical line-broadening (in ppm) showing a chemical shift distribution rather than quadrupolar broadening.^62−64^ This chemical shift distribution thus originates from the variation in the outer co-ordination shells which is due to both a disordered anionic sublattice and displacement of the hydrides from their ideal positions.

To correlate these experimental findings with the DFT models, a simulated ^17^O spectrum was constructed using large, anion disordered cells for the compositions *x* = 0.25 and 1, with a domain size ratio of 3:2 (vide supra; S22), as obtained from the ^1^H spectra (Table 1). The comparison shows a satisfactory correspondence between the experimental and simulated spectra. However, the calculated ^17^O chemical shift distributions are slightly deshielded compared to the experimental values, possibly due to small deviations from the predicted compositions (*x* = 0.25 and 1). Moreover, DFT calculations of SQS structures for a range of x values (S9) show some structure but unfortunately overlap substantially for the different compositions, hindering the determination of specific compositions (*x* value) present in the sample based on a unique range of chemical shifts.

Interestingly, an additional resonance is observed at ∼110 ppm, which is most clearly visible in the spectrum acquired at a spinning speed of 15 kHz (Figure 6a, top). DFT calculations suggest (S12) that this peak belongs to a hydroxide oxygen. Earlier NMR studies have noted that a metal hydroxide oxygen appears to be more shielded than the corresponding oxygen in a metal oxide.^57,65^ Apparently, this happens for the oxides in the oxyhydride lattice as well. The hydroxide peak contribution can be determined quantitatively from the integral by selectively exciting the central transitions^63^ and was found to be nearly 7.2% of the total integral value, as obtained using the quadrupolar fitting tool in ssNake^37^ (S21).

**Figure 7:**
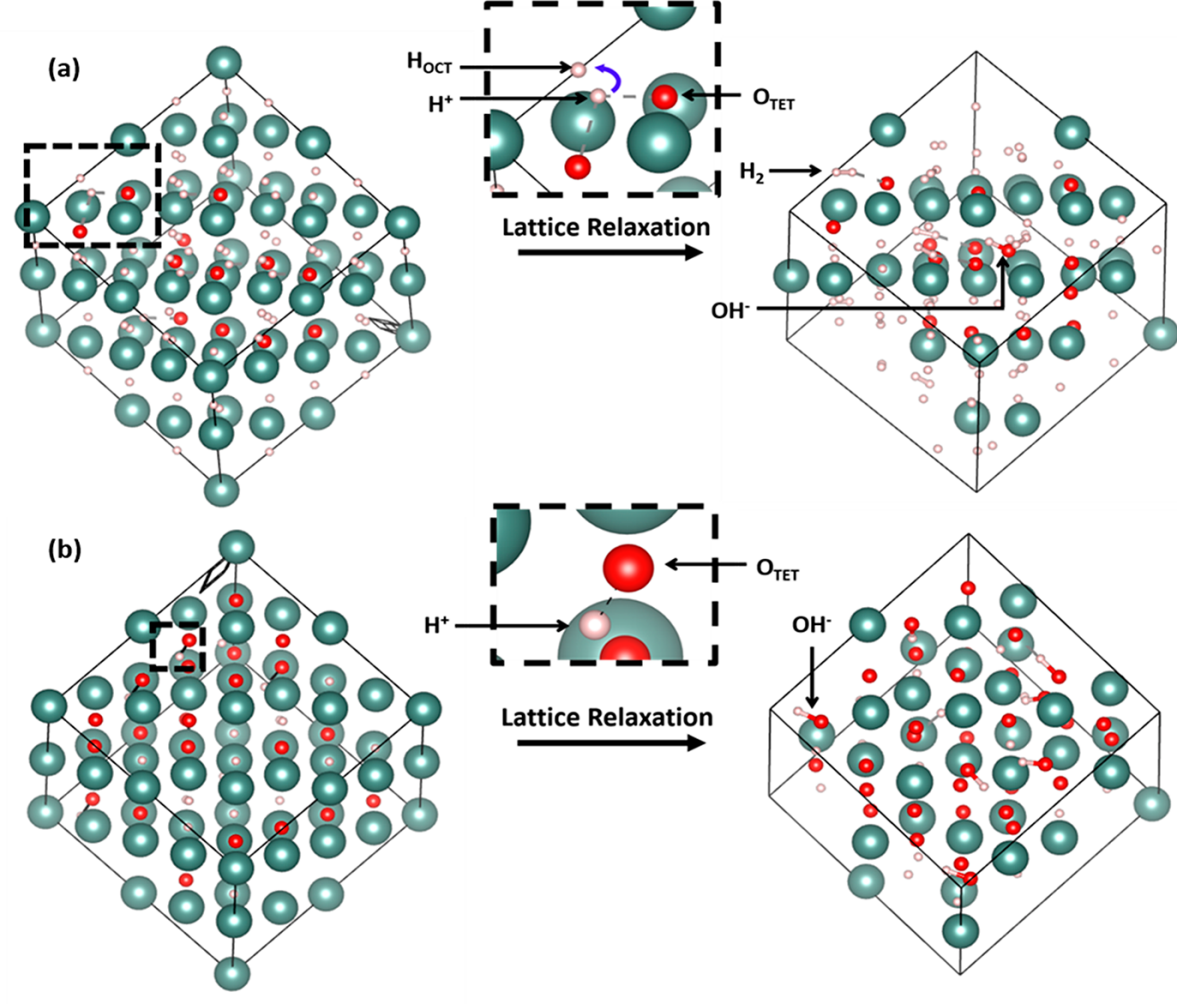
(a). Structural relaxation of a model YO_*x*_H_3–2*x*_ structure (SQS structures), with *x* = 0.25 and 7 OH groups, showing interaction between H^+^ and octahedral H^–^ resulting in 6 H_2_ molecules formed predominantly with only one OH being formed. (b) Structural relaxation of a model SQS YO_*x*_H_3–2*x*_ structure, with *x* = 1 and 6 OH groups showing no H_2_ and 6 OH groups formed due to lack of octahedral H.

**Figure 8:**
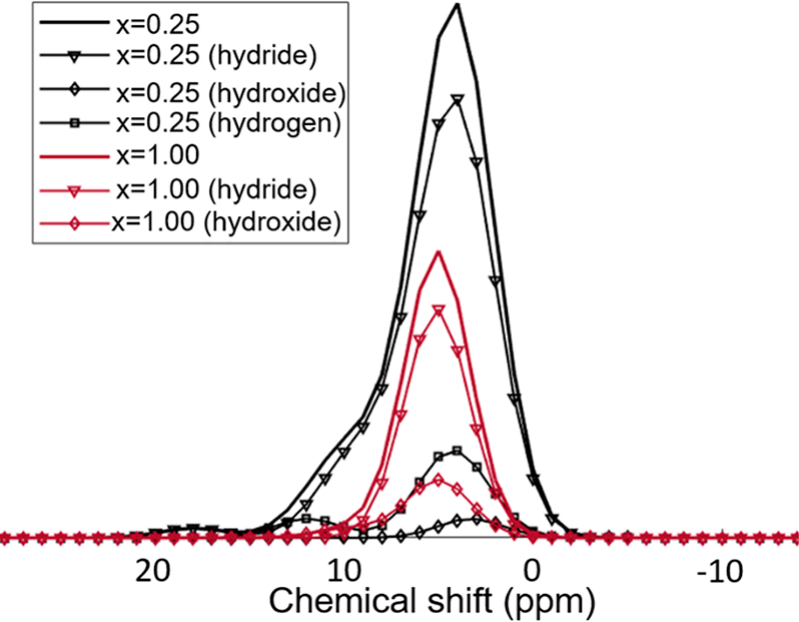
Simulated Gaussian distribution of ^1^H chemical shift for model YO_*x*_H_3–2*x*_ SQS structures, with *x* = 0.25 and 1, including the resonances of the OH hydroxides and H_2_ (for *x* = 0.25).

Based on these results, DFT models with OH^–^ groups in the anion sublattice were constructed for both the hydride-poor and hydride-rich structures (as detailed in S17). In the case of the hydride-poor structure (*x* ∼ 1), all the hydroxide groups were retained after lattice relaxation (Figure 7b), whereas for the hydride-rich structures (*x* ∼ 0.25), the lattice relaxation led mostly to the formation of H_2_ molecules, apart from a few remaining hydroxides. The H_2_ formation mainly occurs in the hydride-rich lattice (*x* = 0.25) due to the presence of nearby octahedral hydrides (Figure 7a), which are absent in the hydride-poor structures (*x* ∼ 1). The presence of OH^–^ and H_2_ has a significant deshielding effect on the ^1^H chemical shift distribution for *x* ∼ 0.25 (Figure 8), which results in a strong overlap of the simulated ^1^H resonances of *x* = 0.25 and 1 compositions. Hence, it substantially resolves the initial contradiction between the calculated and experimentally observed trends for the ^1^H shift as a function of *x* (Figure 3). However, introduction of OH^–^ groups in the lattice might have effects on the overall composition of the oxyhydrides, making it more oxide rich (due to remaining oxides after H_2_ formation). Therefore, we cannot exclude the presence of intermediate compositions, especially in the hydride-rich domain. The hydride-rich domains in the presence of trapped H_2_ (originating from OH^–^ only) might possibly have a composition in the range of 0.25 < *x* < 0.50, while still maintaining a hydride-rich composition. There could, however, be other sources for the formation of molecular H_2_ in the lattice, such that the overall composition does not change. For example, H^+^ from absorbed moisture could combine with H^–^ in the anion lattice to form H_2_. The quantitative prediction of the origin of the observed chemical species is beyond the scope of our current studies. The effect of the OH^–^ incorporation does not have a large impact on the yttrium and remaining oxide chemical shifts (S12 and S13). The H_2_ formation in the model structures matches well with the observation of a narrow peak at 4.6 ppm in the ^1^H NMR spectra (Table-2) and is therefore assigned to trapped H_2_ molecules in the lattice. Indeed, several NMR studies show that trapped H_2_ in metal hydrides is extremely mobile and in many systems occurs at chemical shift values from 4.3 to 5.0 ppm.^8,66−69^

Nevertheless, we discuss other possible assignments of the narrow component (II) in the ^1^H NMR spectrum. As we deduced from the ^17^O spectrum, traces of hydroxide (OH^–^) groups are present; therefore, they should also appear in the ^1^H spectrum. However, their intensity is expected to be very low. The OH^–^ groups have a relatively integrated intensity of 7.2% in the oxygen spectrum. Using the formula YO_*x*_H_(3–2*x*)_ and a 3:2 ratio of domains of composition *x* ∼ 0.25 and 1.0, we can calculate the corresponding percentage of OH^–^ in the ^1^H spectrum to be approximately 2% (S21). This is much less than the relative intensity of 10% of the narrow peak (II) in the proton spectrum. Hence, we can discard the option that this resonance comes from the OH^–^ groups. Moreover, the protons in the hydroxyl groups are expected to be less dynamic, as shown in previous studies,^70−72^ and are therefore less likely to give a very narrow line.

Another possible assignment for peak II is H_2_^–^ formation, as it has been found to be thermodynamically stable in particular cases.^73^ There is insufficient data to identify the properties of such species, however, and hence, its presence remains ambiguous. Therefore, current results for H_2_ formation in the anion lattice are the most likely assignment of peak II.

**Figure 9:**
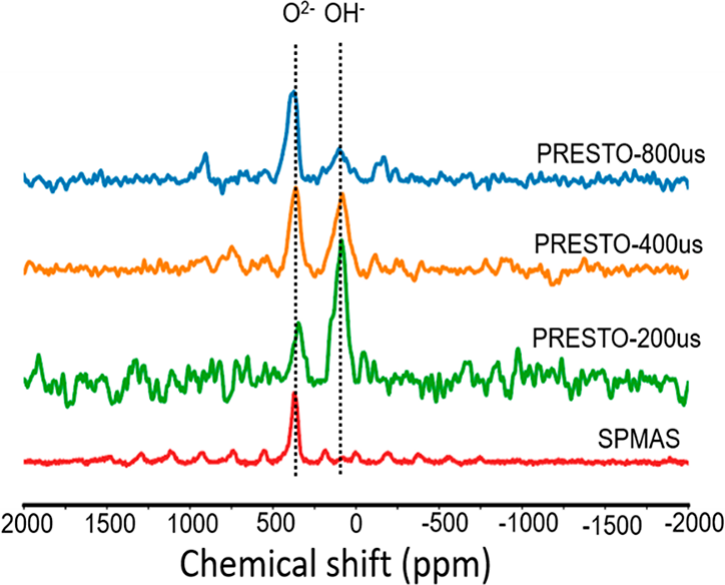
^17^O(^1^H) PRESTO at 15 kHz MAS of yttrium oxyhydride thin film at different recoupling times (80k scans for panels 1, 2, and 4 (from the top); 40k scans in panel 3 rescaled by a factor of 2 for quantitative comparison to the other PRESTO spectra. The spectra were acquired at 14.1 T.

To gain insight into the proximity of different oxygen and hydride species, ^17^O–^1^H PRESTO recoupling experiments were carried out. This pulse sequence is affected by the so-called dipolar truncation effect,^74^ meaning that polarization transfer from ^1^H to distant ^17^O nuclei is attenuated and one therefore mainly probes close proximities, i.e., the ^17^O resonances that are relatively near to ^1^H show a faster buildup of signal intensity. Figure 9 shows the spectra of the ^17^O (^1^H) PRESTO for three different recoupling times (200, 400, and 800 μs). As expected, the hydroxyl oxygen signal (at ∼110 ppm) is very strong already at short recoupling times because of the very close proximity of the hydroxide (OH^–^) proton. For the oxides (O^2–^, between 300 to 475 ppm), we observe that the (convoluted) resonance moves to higher chemical shift values (deshielding) as the recoupling time is increased. This indicates that the more deshielded ^17^O resonances (peak iii, Figure 6b) have a weaker correlation with hydrides than the more shielded ones (e.g., peak i, Figure 6b). In other words, the ^17^O resonances are shifted to higher field (more shielded) as the hydride concentration in their immediate surroundings is larger. This difference in signal build-up for the oxygen resonances substantiates our interpretation that the samples consist of hydride-rich and hydride-poor domains. For a completely disordered anion lattice with a fixed composition, all of the oxides would have the same average dipolar coupling with the hydrides, as the hydride concentrations around them would be similar. Moreover, spin diffusion would be active to distribute the ^1^H polarization uniformly due to strong ^1^H–^1^H homonuclear dipolar couplings, rendering the couplings to ^17^O indistinguishable. This is clearly not the case.

### ^89^Y Solid-State NMR and DFT Calculations

**Figure 10:**
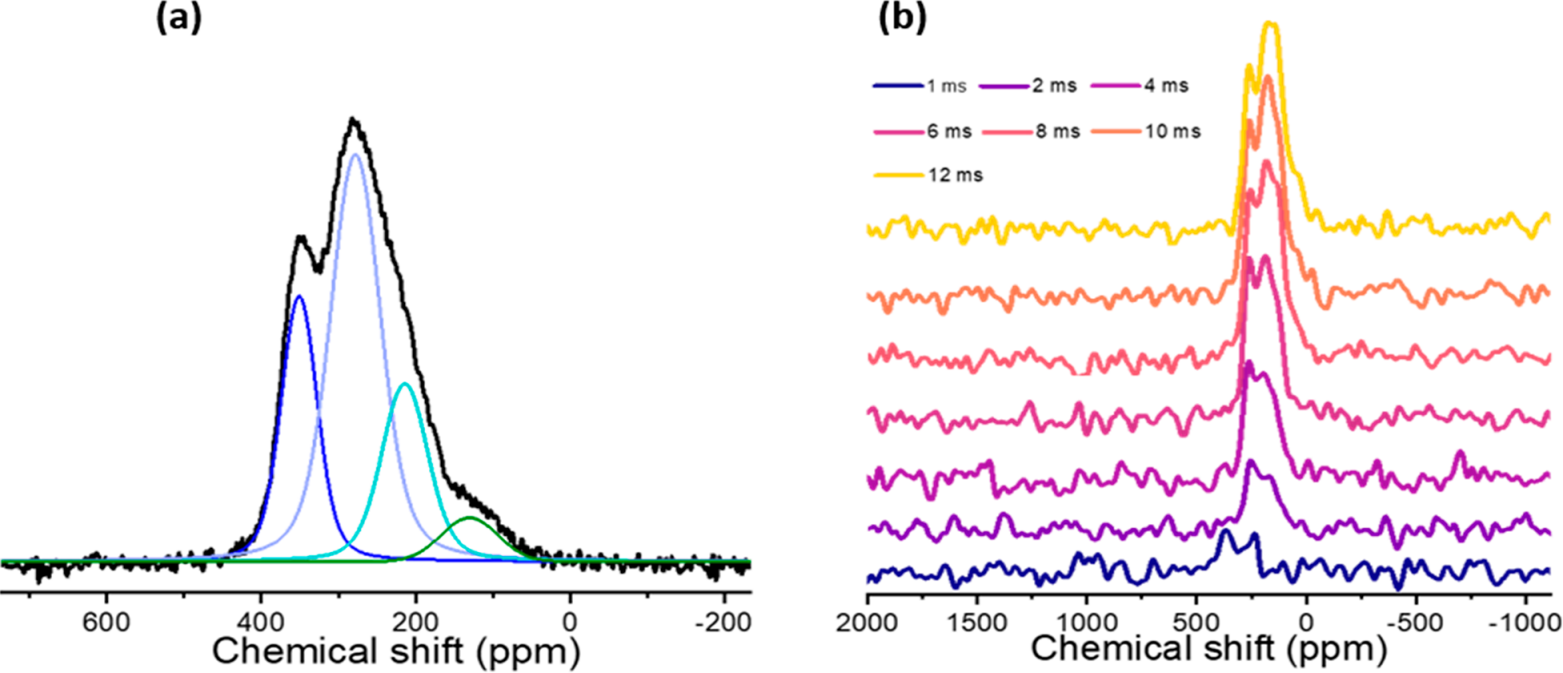
(a) ^89^Y(^1^H) CP-MAS (20 kHz) deconvoluted spectrum of yttrium oxyhydride powder (32k scans) at 8 ms CP contact time. (b) CP-MAS signal intensities for different CP contact times (16k scans). Both spectra were acquired at 20T.

**Figure 11:**
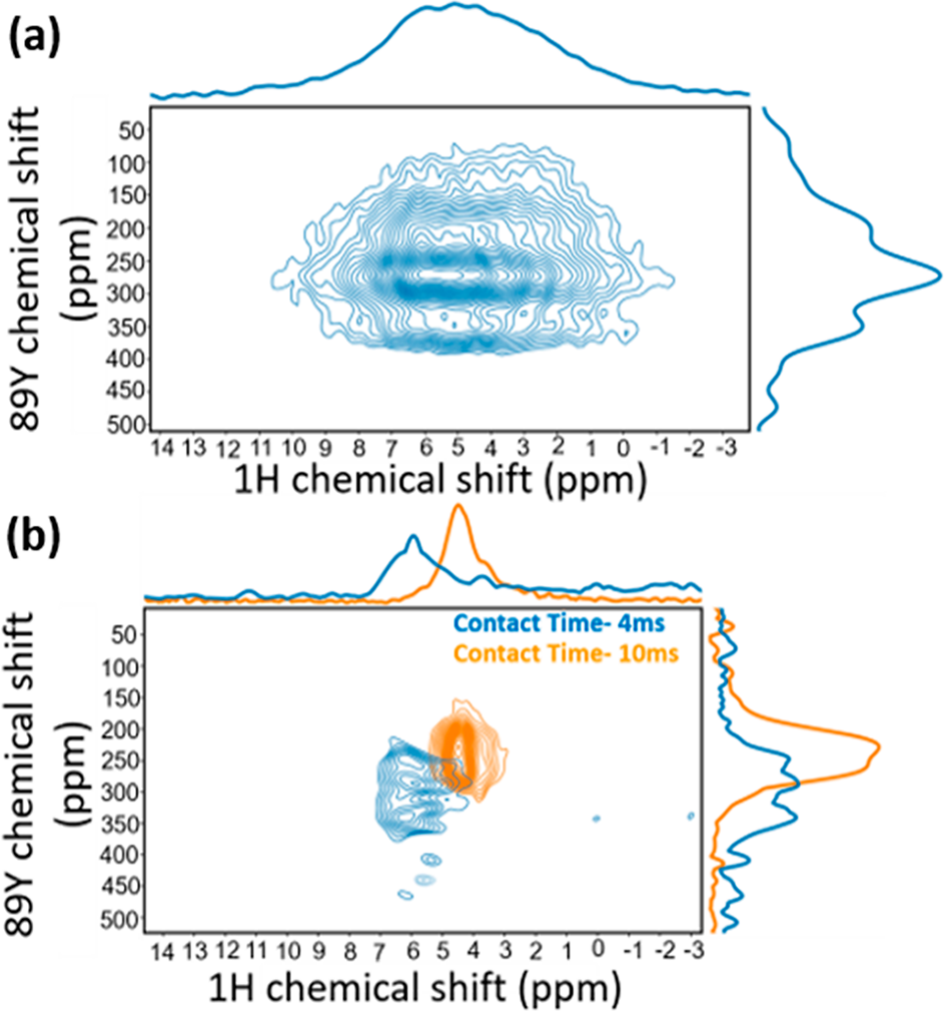
(a) ^1^H–^89^Y inversely detected heteronuclear correlation spectrum of yttrium oxyhydride powder (20 kHz MAS). (b) ^1^H–^89^Y inversely detected heteronuclear correlation spectra obtained using Lee Goldberg cross-polarization (LGCP) with 4 ms (blue) or 10 ms (orange) contact times. The spectra were acquired at 20 T.

Finally, the local environments of yttrium were probed by ^89^Y (^1^H) cross-polarization experiments (Figure 10a). Cross-polarization (CP) uses the heteronuclear dipolar interaction between neighboring spins to transfer polarization. This enhances the signal of nuclei with a low gyromagnetic ratio such as ^89^Y and at the same time provides information about their proximity to nearby protons. The ^89^Y (^1^H) CPMAS spectrum shows four overlapping peaks centered at 350, 275, 210, and 120 ppm, respectively, as extracted from the projection of the ^1^H–^89^Y heteronuclear correlation (HETCOR) spectrum (Figure 11a).

**Figure 12:**
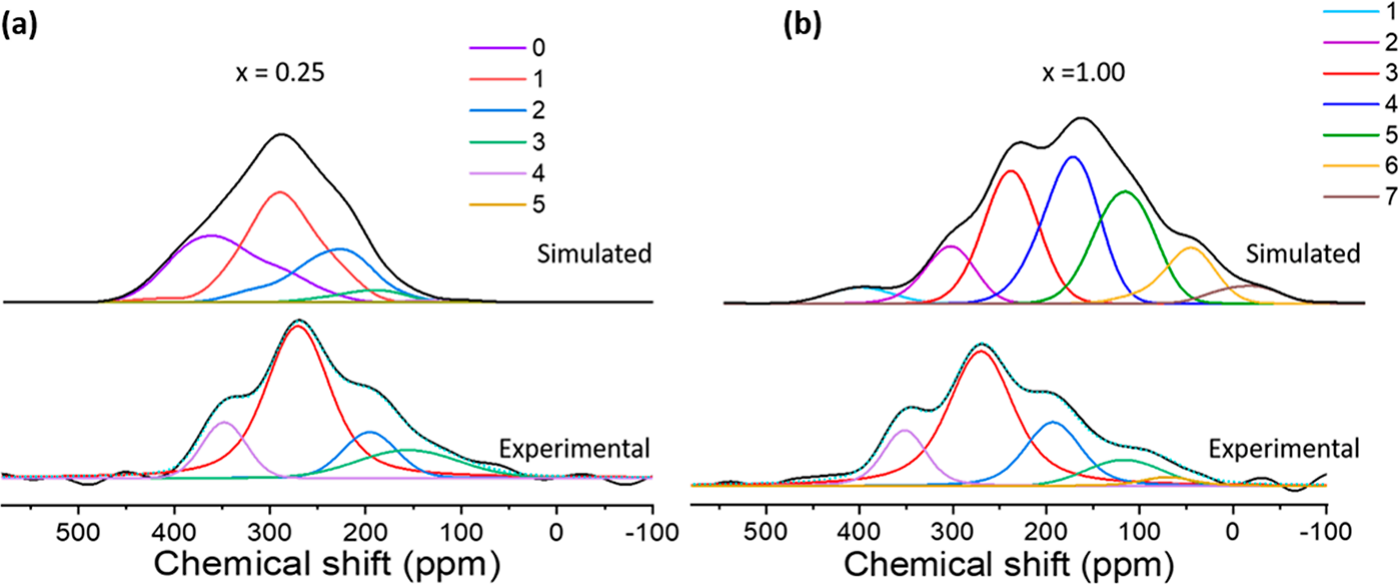
Comparing calculated (top) and experimental (bottom) ^89^Y spectrum of (a) hydride-rich (*x* ∼ 0.25) and (b) hydride-poor (*x* ∼ 1) domains, as a function of oxygen co-ordination. The structures used in the figure are of anion disordered large-cell yttrium oxyhydrides. The simulated peaks are plotted as a function of the number of oxygens in the first co-ordination sphere of yttrium. The two domains show a considerable difference in relative peak intensities in different regions of the ^89^Y spectrum. The hydride-poor domains give a higher intensity in the more shielded (lower ppm) region. The experimental ^89^Y spectra at the bottom are partial projections from the HETCOR spectrum in Figure 11a making projections over the regions of the ^1^H spectrum limited to either the hydride-rich (left) or hydride-poor domains (right). The extracted hydride-rich domain comprises a ^1^H range of 6–8 ppm, and the hydride-poor domain includes a region from 0 to 2 ppm in the ^1^H spectrum as shown in section S24.

The DFT calculations (Figures 12 and S14) of the ^89^Y chemical shifts show a number of effects as a function of composition: (a) For a fixed coordination (i.e., fixed number of oxygens in the first coordination shell), there is a substantial deshielding of Y with increasing *x*. (b) The number of Y-nuclei with a higher coordination obviously increases with *x*. (c) For fixed *x*, the shielding of an yttrium nucleus increases with the number of coordinating oxygens. The net effect is that if x increases, the entire spectrum moves toward lower ppm values (higher shielding). Unfortunately, due to these various effects, it is also not possible to assign specific peaks in the ^89^Y spectrum to specific coordinations or domains with a different composition. The spectrum of different domains consists of various overlapping peaks, and domains with different composition have strongly overlapping spectra (Figure 12a,b). However, it is clear that the yttrium resonances at lower ppm values (higher shielding) experience a weaker coupling to protons, indicating a major contribution from hydride-poor domains. This is apparent from Figure 12, which shows that an increased ^89^Y intensity on the shielded side of the ^89^Y projection over a spectral area corresponding to higher ^1^H shielded area is reflected in DFT calculations for a higher *x*. The yttrium resonances at higher ppm values are correlated more prominently to the deshielded ^1^H spectral area, which indicates that these resonances have a higher contribution from the hydride-rich domains; again, this is reflected in the DFT calculations as well.

We can also extract information about the existence of domains with different compositions by looking at the efficiency of the cross-polarization (CP) process between ^89^Y and the protons in the sample. The evolution of the CP signal intensity with the contact time is characterized by a build-up that is determined by the strength of the dipolar interaction (i.e., distance and number of spin pairs involved) with a subsequent (*T*_1ρ_) decay that depends on dynamics in the kHz regime.^75^ As shown in Figure 10b, the ^89^Y CP signal builds up more slowly for the more shielded peaks (centered at 210 and 120 ppm) compared to the less-shielded peaks (centered at 350 and 275 ppm). The CP build-up is slower for regions containing less hydrides (i.e., a hydride-poor region; *x* ∼ 1) compared to hydride-rich domains (*x* ∼ 0.25). As described above the DFT calculations show that yttrium nuclei in the hydride-rich domains are overall more deshielded (higher chemical shift values), hence, the faster signal build-up in the deshielded region of the CP spectra. Despite the strong overlap of the spectra of different domains, the relative contributions are different on moving from a more shielded region to a less shielded region (see Figure 10b). This confirms the observations from the 2D HETCOR spectrum described above extracting ^89^Y projections over the ^1^H shift region from the hydride-poor and hydride-rich domains (Figures 12 and S24).

To even further strengthen this analysis, 2D ^1^H–^89^Y HETCOR spectra, using Lee Goldburg CP, were obtained at different (4 and 10 ms) LGCP contact times (Figure 11b). At shorter contact times, signals from strongly dipolar coupled nuclei dominate, whereas at longer contact times signals with a weaker dipolar coupling become more prominent. We see a clear shift from the deshielded to the shielded region for both the ^89^Y and ^1^H shifts, corresponding to the hydrogen rich and poor domains, respectively, in line with the prediction by the DFT calculations for ^89^Y. The fact that the more shielded proton resonances correspond to the hydride-poor domains is contrary to the initial DFT calculations, but explainable when the effects of OH^–^ incorporation were taken into account (vide supra).

## Conclusion

In summary, the NMR experiments and DFT calculations provide new insights in the structure of yttrium oxyhydride, in particular, regarding the arrangement of the heteroanions. The presence of hydride-rich and -poor domains was established. The domain formation could be a result of unequal pore sizes in the yttrium hydride films, leading to different concentrations of oxygen incorporated in different regions of the thin films upon exposure to air/oxygen. Moreover, the thickness of the films (1 μm in this work) might contribute to the observed compositional variation, but a direct correlation cannot be established from the current studies. Although we modeled our experiments mostly as a binary system of compositions *x* ∼ 0.25 and 1, we cannot rule out the presence of intermediate compositions, especially for the hydride-rich domains. Further insight regarding the anion arrangement might be gained from knowledge of the precise domain sizes, which was not studied in detail here. Nevertheless, the presence of hydride-rich and -poor domains strongly indicates a lower propensity for complete mixing of the hydrides and the oxides, which could be due to the contrasting nature of the two anions in the multianion lattice. Note that since we deposit a dihydride, the formation of an *x* ∼ 0.25 compound requires the addition of hydrogen to the lattice during exposure to air. Alternatively, it could suggest phase segregation on oxygenation. Previously, Hans et al.^76^ have reported the occurrence of dual phases in gadolinium oxyhydride thin films, where they noted the presence of Gd_2_O_3_ and GdH_2_ regions. Here we do not observe such drastic compositional variation, as the ^89^Y and ^2^H NMR do not show any evidence of presence of metallic YH_2_.^54,77^

The combination of ^17^O NMR and DFT modeling proves to be an important tool in understanding not only the environments and oxidation state of the oxides but also allowed extraction of the oxidation states and different chemical species of hydrogen. Quantitative studies on the presence of both neutral (H_2_) and hydroxide (OH^–^) hydrogen species help in the assignment of the remaining ^1^H peaks as hydrides (H^–^). The concentration of the trapped H_2_ in the lattice may have significant influence on the dynamics and efficiency of the photochromic nature of the materials, as our previous study showed that a mobile fraction of the H atoms plays an important role in the photochromism.^26^

The presence of hydroxyl groups and trapped hydrogen in the lattice was established and quantified by ^1^H and ^17^O NMR studies. The separation of the ^17^O and ^1^H spectra for the hydride-rich and hydride-poor domains could not be realized because of the substantial overlap of the chemical sites of the regions owing to the complexity of the anionic arrangement. Although showing a lot of overlap, ^89^Y spectra for both domains were extracted, and an agreement with the simulated ^89^Y spectra was obtained. ^1^H and ^2^H NMR studies, along with DFT calculations, are combined to study hydride ion dynamics, which show minimal or no dynamics for the hydrides, whereas the trapped hydrogen displays very high mobility.

The mechanism for photochromism is far from settled. At its heart is the electron–hole pair generated by the bandgap excitation.^81^ Presumably, the excited electron reduces the RE^3+^ while the hole oxidizes the H^–^-ion, in particular the hydrogen ion at the octahedral vacancy seems prone to this.^82^ Probably some (filamentary) clustering of the reduced rare earth metal takes place, which causes the optical absorption.^50,83^ However, the nature and the role of the oxidized H^–^ are still unclear. Solid-state NMR studies of more suitable metal oxyhydrides could be useful in getting more insight into this phenomenon. We are currently pursuing solid state NMR of scandium oxyhydride thin films for such studies. As ^45^Sc is 100% abundant and has a higher gyromagnetic ratio than ^89^Y, it is favored in terms of sensitivity.

## Supporting Information Available

Solid-state NMR experimental details; Optical transmission measurements; ^17^O PRESTO reference measurements; Ordered yttrium oxyhydride model polymorphs; Yttrium oxyhydride thin film *T*_2_ measurements; ^1^H line width measured at different magnetic fields; ^2^H low temperature measurements; ^1^H calculated chemical shift values for different *x* values in YO_*x*_H_(3–2*x*)_ (ordered and SQS; before and after lattice relaxation); ^17^O DFT calculations of SQS structures (quadrupolar parameters and chemical shifts); DFT calculations of model yttrium oxyhydride structure (ordered) with octahedral O; ^17^O SP-MAS at different magnetic fields; ^89^Y chemical shift trends in YO_*x*_H_(3–2*x*)_ SQS structures; Modeling O–H incorporated SQS YO_*x*_H_(3–2*x*)_; Matlab scripts; Comparing simulated and experimental ^17^O spectrum for SQS YO_*x*_H_(3–2*x*)_; ^1^H quantitative studies of yttrium oxyhydride thin films; Calculated total energies of various compositions of yttrium and lanthanum oxyhydrides (SQS and random models); DFT+U chemical shift calculations (PDF)
