# Supplementary material for: Exploring Multi-Anion Chemistry in Yttrium Oxyhydrides: Solid-State NMR Studies and DFT Calculations
Source: J Phys Chem C Nanomater Interfaces. 2023 Jul 17;127(29):14303–16. doi: 10.1021/acs.jpcc.3c02680 (PMC10388355; doi:10.1021/acs.jpcc.3c02680)
Supplement: Supplementary file 1 — jp3c02680_si_001.pdf [file jp3c02680_si_001.pdf]

Exploring Multi-Anion Chemistry in Yttrium Oxyhydrides: Solid-state NMR Studies and DFT Calculations

Shrestha Banerjee[1], Diana Chaykina[2], Rens Stigter[3], Giorgio Colombi[2], Stephan W.H. Eijt[3], Bernard Dam[2], Gilles A. de Wijs*[1], and Arno P.M. Kentgens*[1]

[1]Radboud University, Institute for Molecules and Materials, Heyendaalseweg 135, NL-6525 AJ Nijmegen, The Netherlands.

[2]Materials for Energy Conversion and Storage, Department of Chemical Engineering, Delft University of Technology, Van der Maasweg 9, NL-2629 HZ Delft, The Netherlands.

[3]Fundamental Aspects of Materials and Energy, Department of Radiation Science and Technology, Faculty of Applied Sciences, Delft University of Technology, Mekelweg 15, NL-2629 JB Delft, The Netherlands.

CONTENTS

S1: Optical transmission measurements S3
S2: ^{17}O PRESTO reference measurements: L-Tyrosine S4
S3: Ordered yttrium oxyhydride model polymorphs S5
S4: Yttrium oxyhydride Hahn echo array (T_2 measurements) S6
S5: Comparing ^{1}H linewidth measured at different magnetic fields S7
S6: ^{1}H chemical shift values (DFT calculations) of an SQS structure S8
S7: ^{2}H low temperature measurements S9
S8: ^{1}H calculated chemical shift values for different *x* in $YO_xH_{(3-2x)}$ (lattice edge length = 5.23Å) S10
S9: ^{17}O DFT calculations of SQS structures S11
S10: Ordered model yttrium oxyhydride structurewith octahedral O (lattice edge length = 5.23Å) S12
S11: ^{17}O SPE-MAS at different magnetic fields S13
S12: ^{17}O spectrum (DFT calculations) of model SQS $YO_xH_{(3-2x)}$ at x=0.25 (lattice edge length = 10.65Å) S14
S13: Calculated ^{89}Y spectrum of model SQS $YO_xH_{(3-2x)}$ at x=0.25 with (lattice edge length = 10.65Å) S15
S14: ^{17}O deconvoluted spectrum of Yttrium oxyhydride thin film S17
S15: Table S1 Calculated quadrupolar parameters (C_Q and η) for ^{2}H and ^{17}O in $YO_xH_{(3-2x)}$
S16: SQS structures with x=0.25 and x=1 (edge length= 10.65Å). S18
S17: ^{1}H calculated chemical shift after O-H incorporation into the model SQS $YO_xH_{(3-2x)}$ (edge length= 10.65Å) S19
S18: Matlab scripts used for simulated gaussian distribution for calculated H, Y and O chemical shifts S20
S19: Solid State NMR experimental details: S21
S20: Table S2 Comparing the large and small $YH_xO_{(3-2x)}$ cell NMR parameters S22
S21: Deconvolution of the ^{17}O MAS spectrum (15kHz spinning frequency) with the O-H group S23
S22: Comparing simulated and experimental ^{17}O spectrum for SQS $YO_xH_{(3-2x)}$ S24
S23: ^{1}H Quantitative studies of Yttrium oxyhydride thin films (empirical formula determination) S25
S24: Extraction of ^{89}Y spectra of hydride rich (x=0.25) and hydride poor (x=1) domains ... S26
S25: Calculated total energies for various compositions of yttrium and lanthanum oxyhydrides (SQS and random models) S27
S26: DFT + U chemical shift calculations S28

S1: Optical transmission measurements

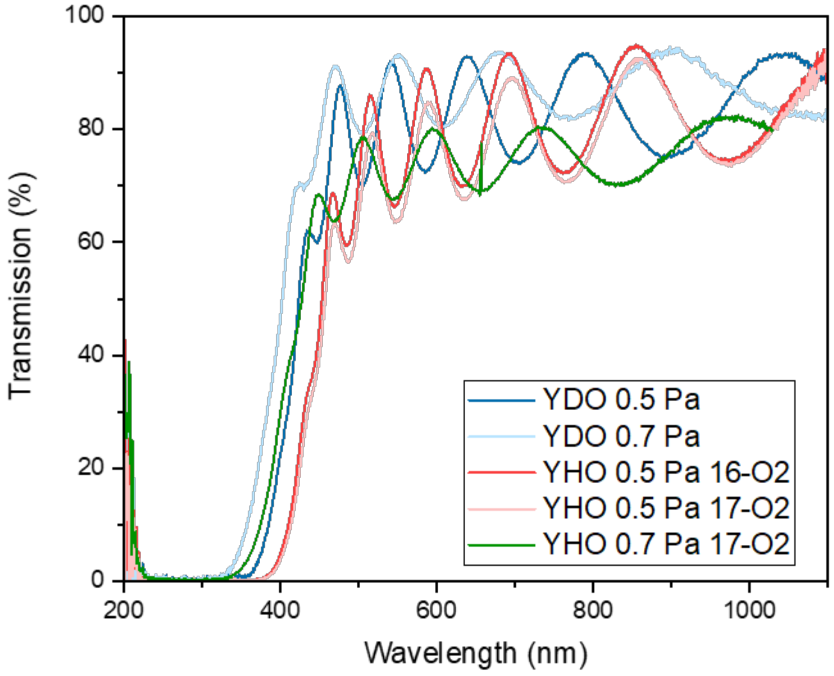

Figure S1: Optical transmission spectra for yttrium oxyhydride thin films deposited at different H2 (Ar) pressures.

Optical transmission spectra were measured for several yttrium oxyhydride thin films on quartz substrates (TedPella, 10x10 mm2) at room temperature (21 °C) using a custom-built optical fiber spectrometer containing a deuterium and a quartz tungsten halogen lamp (DH2000-BAL, Ocean Optics B.V.), and two Si array wavelength-dispersive spectrometers (HR4000 and Flame, Ocean Optics B.V.). Results show energy band gap of nearly 3.09 eV (400nm), as optical transmission is zero until this regime.

S2: ^{17}O PRESTO reference measurements: L-Tyrosine

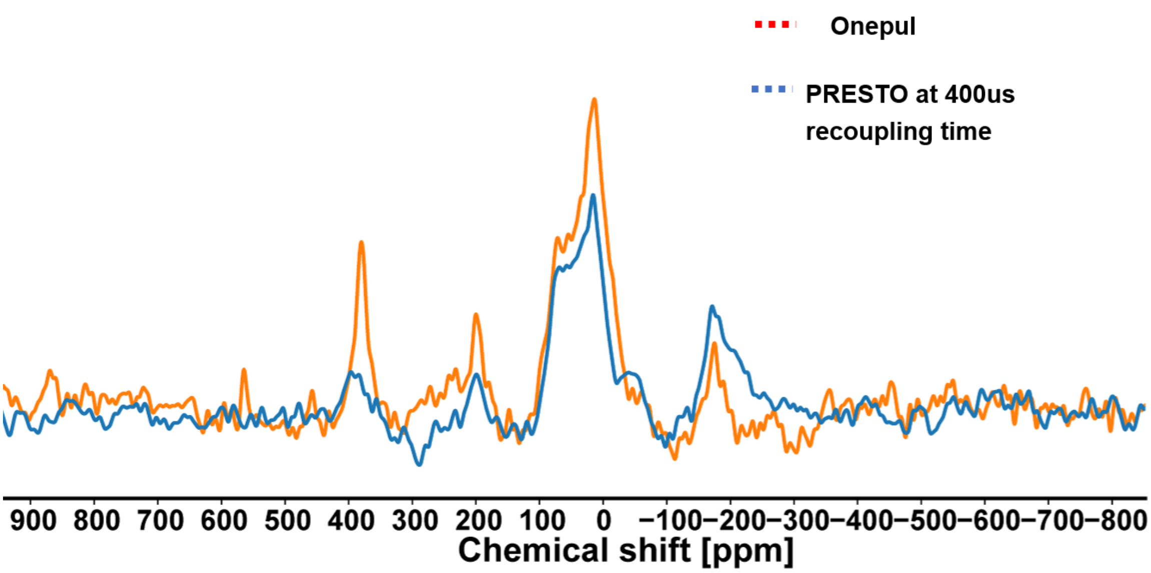

Figure S2: L-Tyrosine with 35-40 % ^{17}O dilution of the hydroxyl group

L-Tyrosine with 35-40 % ^{17}O dilution of the hydroxyl group was used for measurement setup. The PRESTO experiments were done under similar conditions as Yttrium oxyhydride thin films (Number of scans =10k; less than YHxOy thin films) and is compared with the Single pulse excitation (SPE) spectrum of ^{17}O. The ratio of SPE:PRESTO is 1.68.

S3: Ordered yttrium oxyhydride model polymorphs

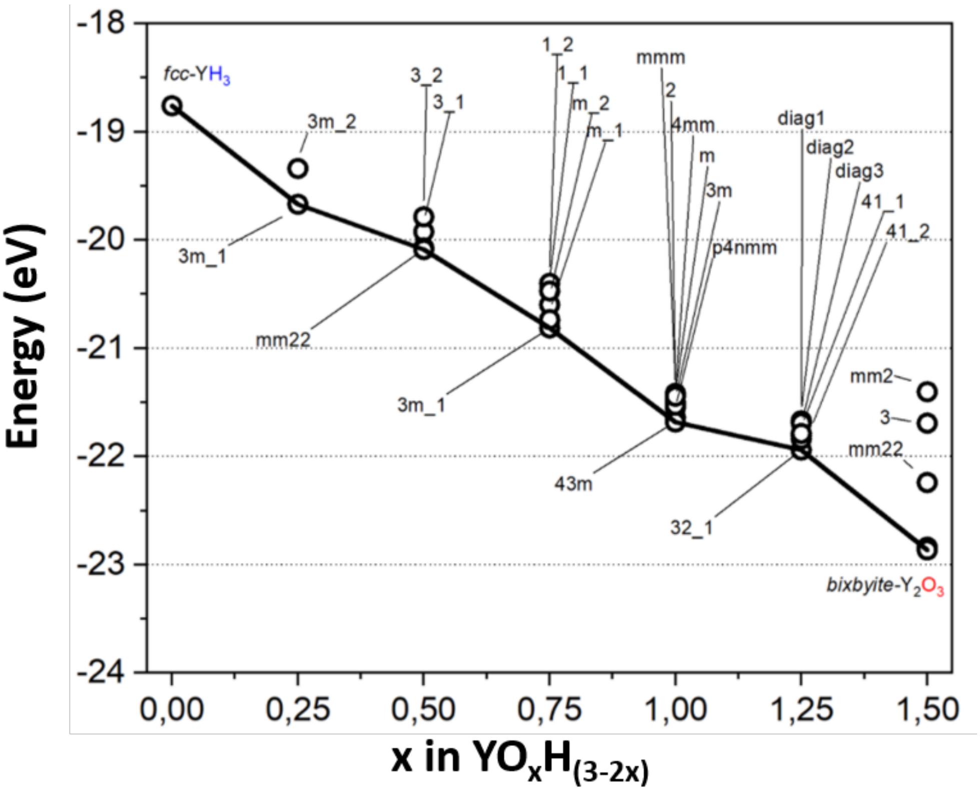

Figure S3: The symmetry elements for the different compositions (x values) of yttrium oxyhydride structures with anion-ordered sub-lattice.

For x= 1, 1.25, and 1.5, the names reflect the elements of symmetry that can be found in the FCC cell. For x = 1.25 only, there are three structures without any elements of symmetry that are called diag1, diag2, and diag3. For x < 1.0 the names only refer to the symmetry of the tetrahedral atoms. To distinguish cells that have the same tetrahedral symmetry but with H in different octahedral positions, incremental numbers *_1, *_2 are used.

S4: Yttrium oxyhydride Hahn echo array (T_2 measurements)

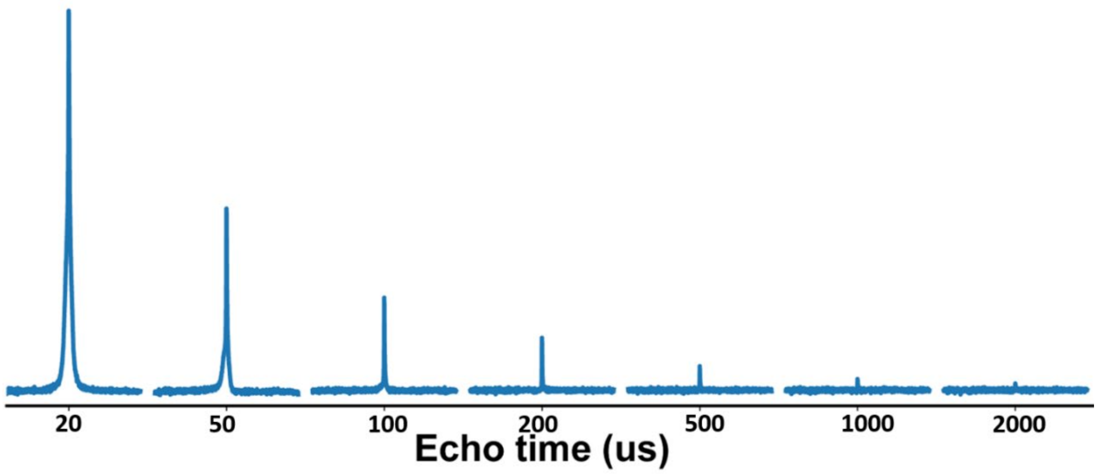

Figure S4: Static ^1H T_2 (spin-spin relaxation time) measurements of yttrium oxyhydride thin film acquired using proton free probe at 300MHz spectrometer.

Static ^1H T_2 (spin-spin relaxation time) measurements show that the narrow component has a long T_2 This is attributed to mobility of the H species. The T_2 of the narrow component is ~250us and that of the broad components is ~30us as obtained by fitting the above spectra with T_2 relaxation equation using ssNake.

S5: Comparing ^{1}H linewidth measured at different magnetic fields

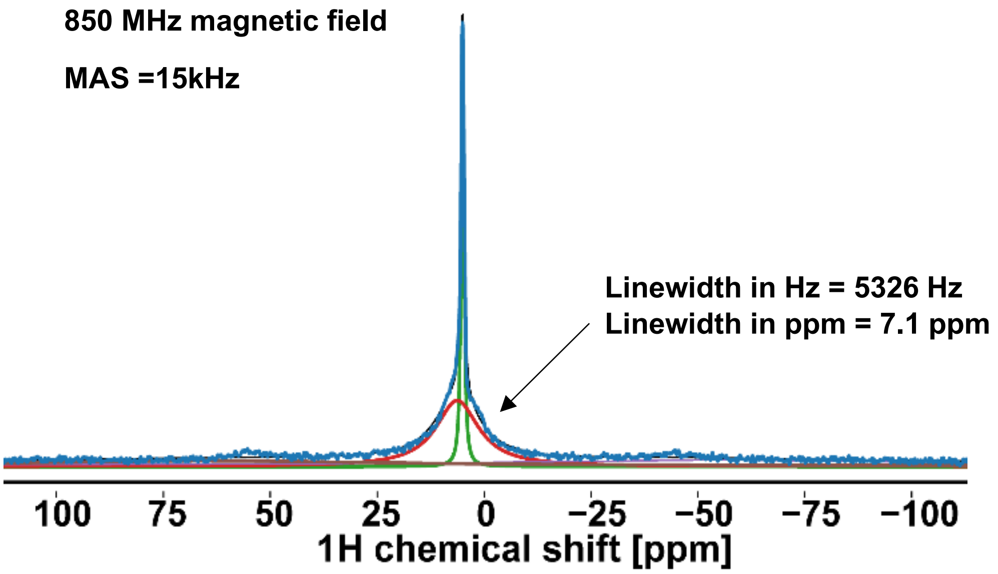

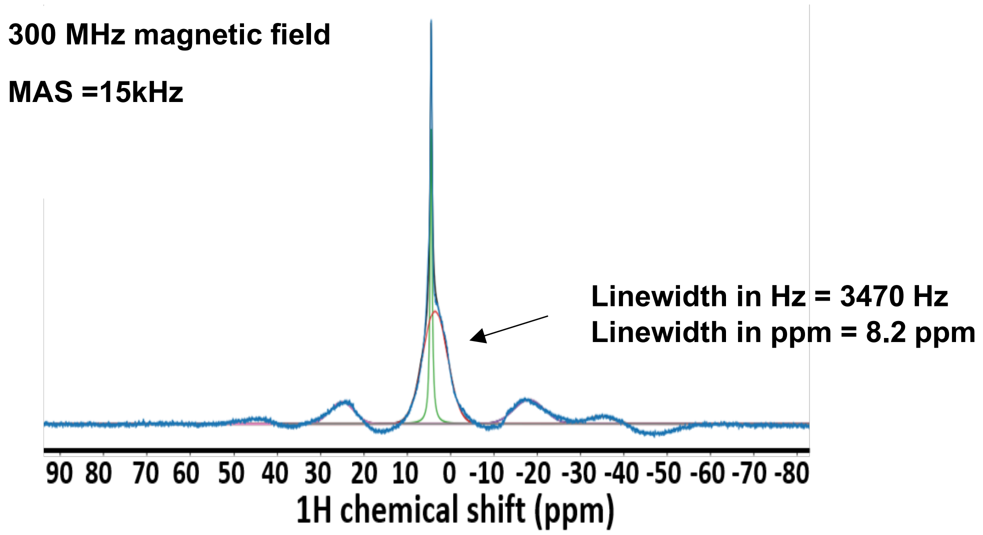

Figure S5: (top) 1H yttrium oxyhydride thin film spectra (H_2 dep. pressure 0.5 Pa) acquired at 850 MHz with 15kHz MAS, (bottom) 1H yttrium oxyhydride thin film spectra acquired at 300 MHz with 15kHz MAS.

The linewidth of the broad component of the ^{1}H spectra (spinning frequency 15kHz) for YH_xO_y powder sample compares well in ppm units and appears to be distinctly different in Hz units. This implies, that the ^{1}H linewidth is predominantly determined by a chemical shift distribution (linewidth in ppm) rather than ^{1}H-^{1}H dipolar coupling as residual dipolar coupling which is independent of the external magnetic field strength.

S6: <u>^{1}H chemical shift values (DFT calculations) of an SQS structure</u>

1H chemical shift before relaxation

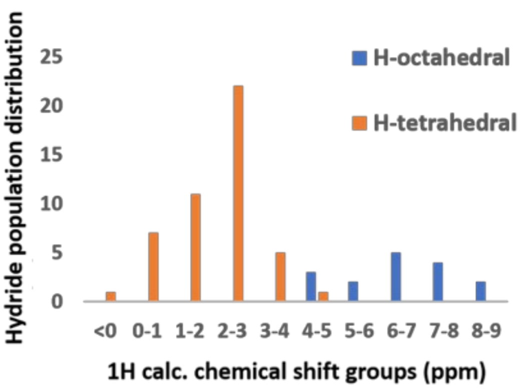

1H chemical shift after relaxation

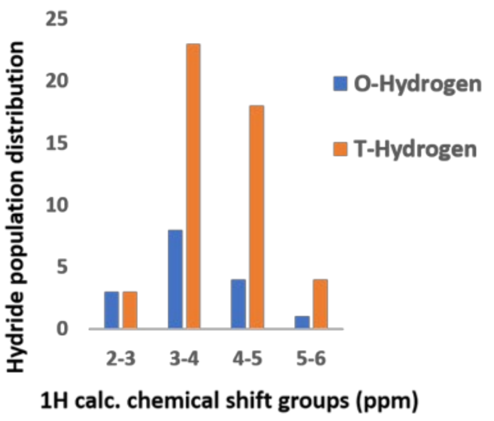

Figure S6: The histograms show the distribution of the 1H calculated chemical shifts of yttrium oxyhdride SQS structure before and after lattice relaxation.

^{1}H chemical shift calculations using VASP of a quasi-random structure show that the octahedral and tetrahedral hydrides overlap less when they are not relaxed. After lattice relaxation they are displaced from the ideal octahedral and tetrahedral sites to intermediate lattice positions and are then distributed over an overlapping range of chemical shift values. Therefore they are no longer distinguishable by their chemical shift in the NMR spectra.

S7: ^{2}H low temperature measurements

The low temperature SPE MAS (10kHz) ^{2}H NMR measurements show no change in the linewidth, indicating there is minimal influence of motion for the hydrides in the lattice. Some minor changes however, can be noted for the quadrupolar coupling constant, which does not confirm presence of ^{1}H mobility, as this can occur because of changes in lattice dimensions at lower temperatures. High temperature measurements were not included in this study, as the materials degrade by evolving hydrogen upon heating.

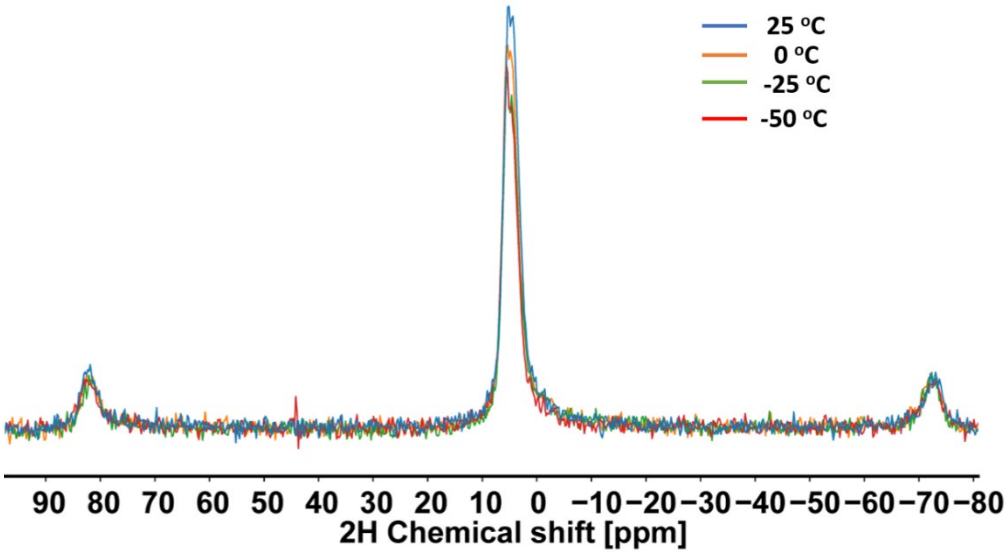

Figure S7: The figure shows an overlay of ^{2}H-MAS yttrium oxyhydride thin film spectra (H_2 dep. pressure 0.5 Pa) measured at variable temperatures.

S8: [1]H calculated chemical shift values for different *x* in $YO_xH_{(3-2x)}$ (lattice edge length = 5.23Å)

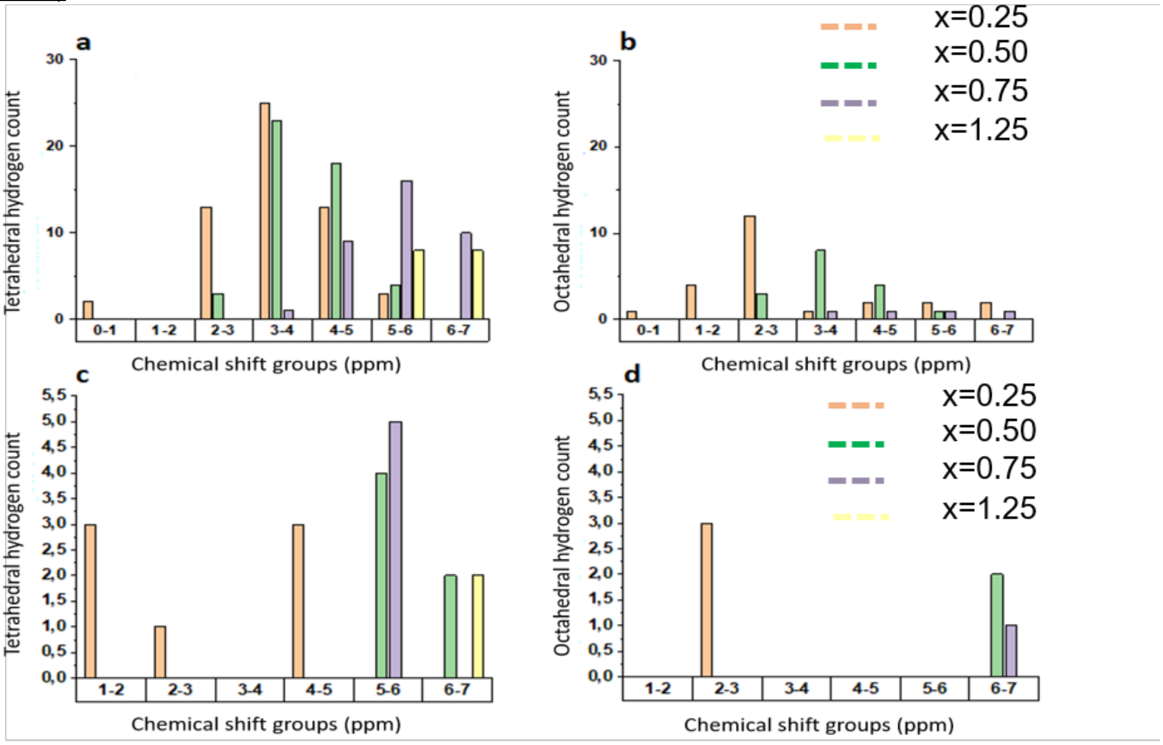

Figure S8: Calculated ^{1}H chemical shift distribution (after lattice relaxation) for (a) Special Quasi-random structures-tetrahedral hydrogen (b)Special Quasi-random structures-octahedral hydrogen (c) Ordered structures- tetrahedral hydrogen (S-3) (d) Ordered structures- octahedral hydrogen (S-3)

The calculations shows that disordered yttrium oxyhydride SQS structures show a substantial distribution in chemical shift for all compositions (x values) whereas the ordered structure show distinct chemical shifts for different hydrides in the structure.

S9: ^{17}O DFT calculations of SQS structures

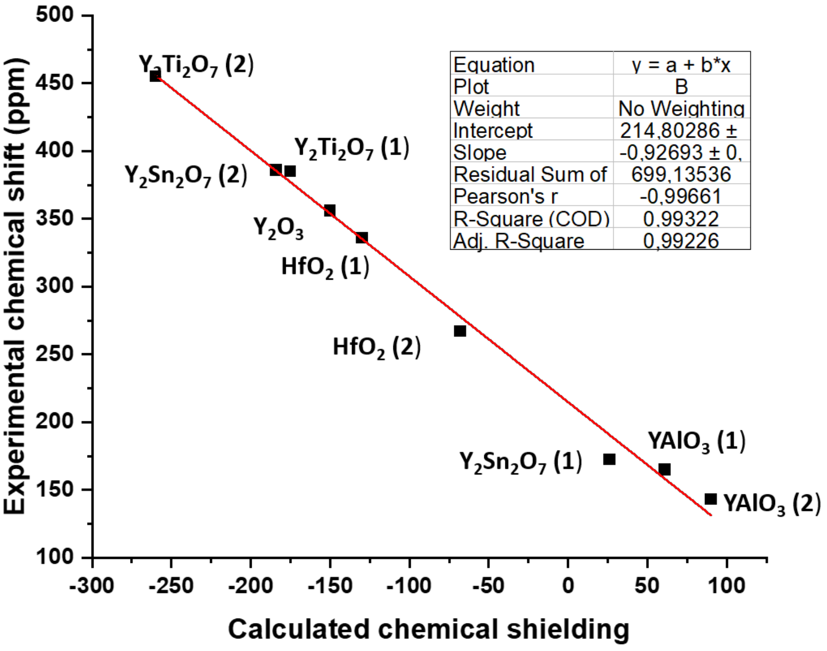

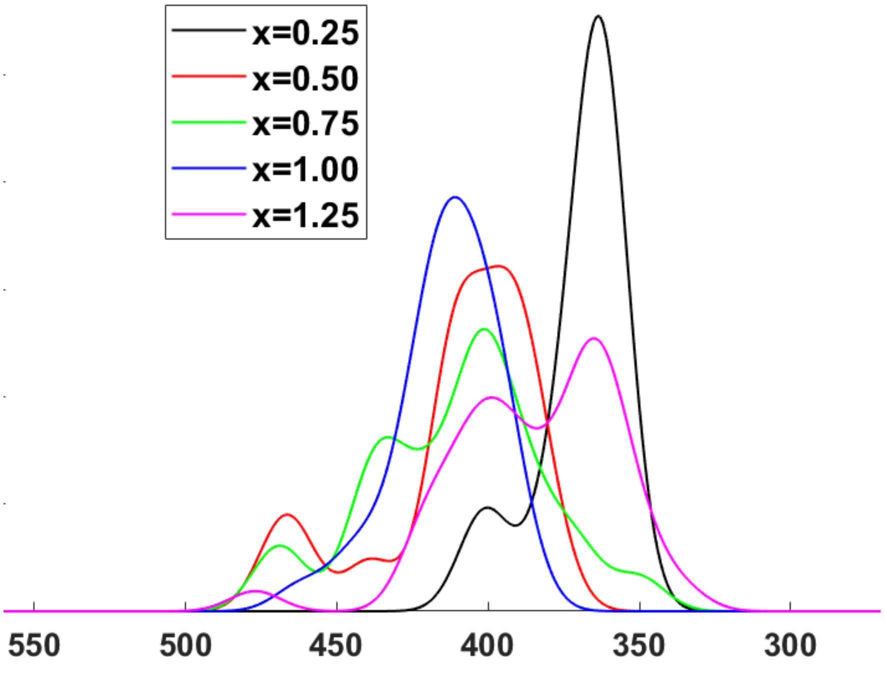

Figure S9: (top) ^{17}O DFT calculations of model compounds, (bottom) chemical shift distribution of model $YO_xH_{(3-2x)}$ with disordered anion sub-lattice (SQS structure after lattice relaxation; lattice edge length = 10.65Å)

The bottom figure shows a lot of overlap of the chemical shifts for different compositions. The simulated spectra are constructed from the calculated shifts using the Matlab script in S-18 using a Gaussian linewidth of 20 ppm for each data point obtained from DFT calculations.

S10: Ordered model yttrium oxyhydride structurewith octahedral O (lattice edge length = 5.23Å)

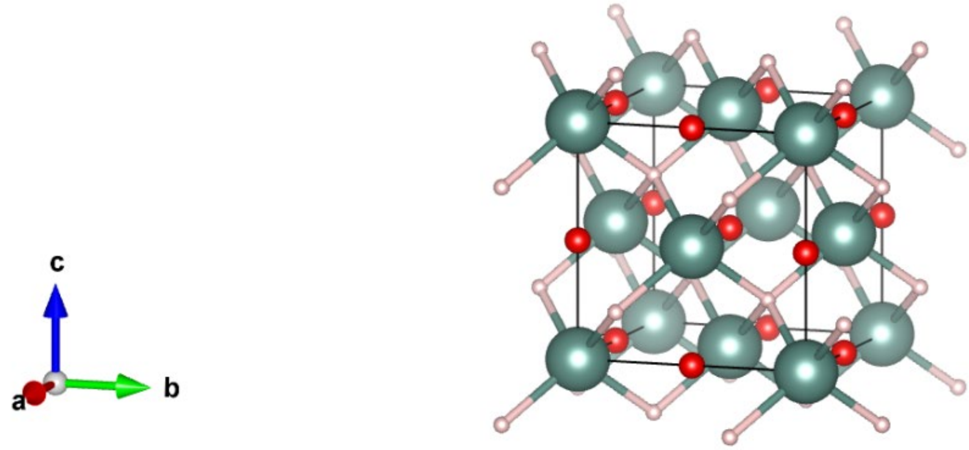

Nuclei	Calculated chemical shift (ppm)
^{89}Y (green spheres)	148
^{1}H (white spheres)	0.61
^{17}O (red spheres)	248.32

Figure S10: The YHO (x=1) structure shows octahedral occupancies of oxygen (red spheres) and tetrahedral occupancies of hydrogen (white spheres) in the FCC yttrium (green spheres) lattice. The calculated chemical shift of the oxide ions in this structure is 248 ppm, which does not comply with the experimental results, implying that the real structures are disordered.

S11: <u>^{17}O SPE-MAS at different magnetic fields</u>

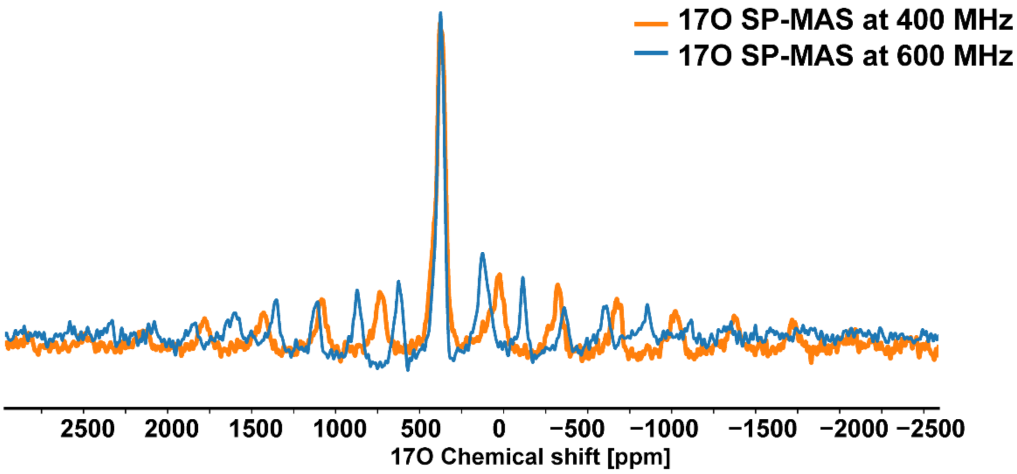

Figure S11: Overlay of two ^{17}O MAS spectra of ^{17}O-labelled yttrium oxyhydride thin films (H_2 dep. pressure 0.5 Pa) at different magnetic fields.

The central transition has a very similar overall linewidth in ppm indicating that this is determined by a chemical shift distribution rather than a second order quadrupolar broadening which has an inverse relationship with the external magnetic field strength.

S12: ^{17}O spectrum (DFT calculations) of model SQS $YO_xH_{(3-2x)}$ at x=0.25 (lattice edge length = 10.65Å)

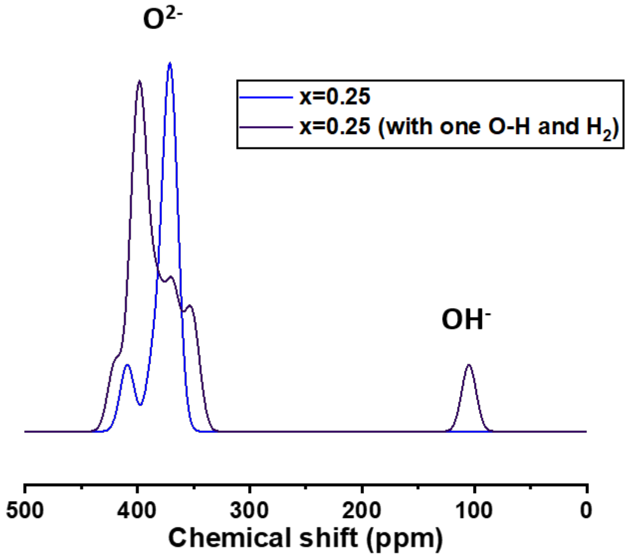

Figure S12: Overlay of two simulated spectra: ^{17}O spectrum for x=0.25 SQS (blue) and ^{17}O spectrum for x=0.25 SQS with inserted O-H groups (as detailed in S-21).

We see clearly the O-H peak at 110 ppm in the calculated spectrum. The simulated spectra are constructed from the calculated shifts using the Matlab script in S-18, using a line broadening of 8 ppm.

S13: Calculated ^{89}Y spectrum of model SQS $YO_xH_{(3-2x)}$ at x=0.25 with (lattice edge length = 10.65Å)

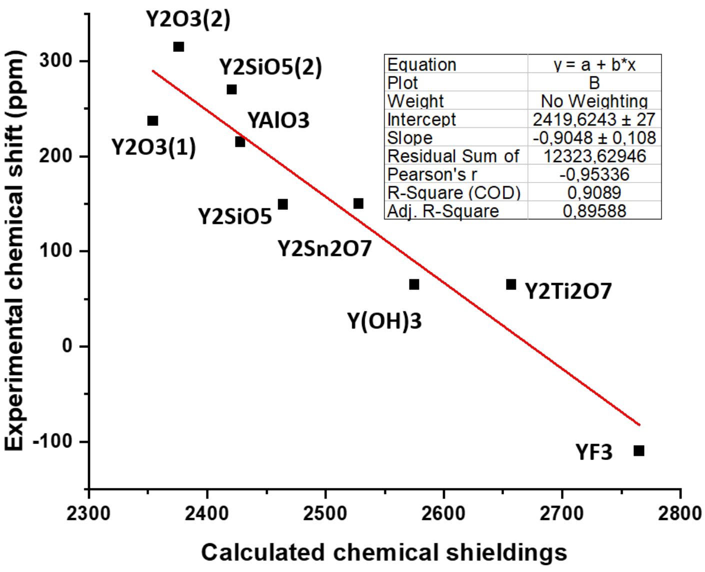

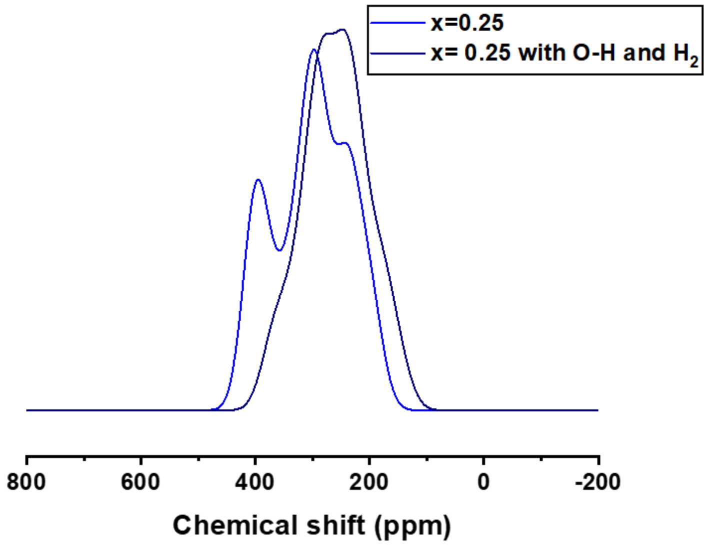

Figure S13: (top) Regression plot for ^{89}Y chemical shifts, correlating the calculated and experimental chemical shifts of a number of reference compounds, (bottom) overlay of the calculated ^{89}Y spectrum of an SQS structure with x=0.25 and the ^{89}Y spectrum of the same structure with inserted O-H groups (detailed in S-21).

We see clearly that chemical shift range of the structure containing OH groups shifts to higher shielding. However, the shift of about 25ppm is small when compared to the overall shift range (300ppm) and hence does not cause substantial difference in the visible trends. The simulated spectra are constructed from the calculated shifts using the Matlab script in S-18. using a linewidth of 20 ppm.

S14: ^{89}Y chemical shift trends in $YO_xH_{(3-2x)}$ disordered structures

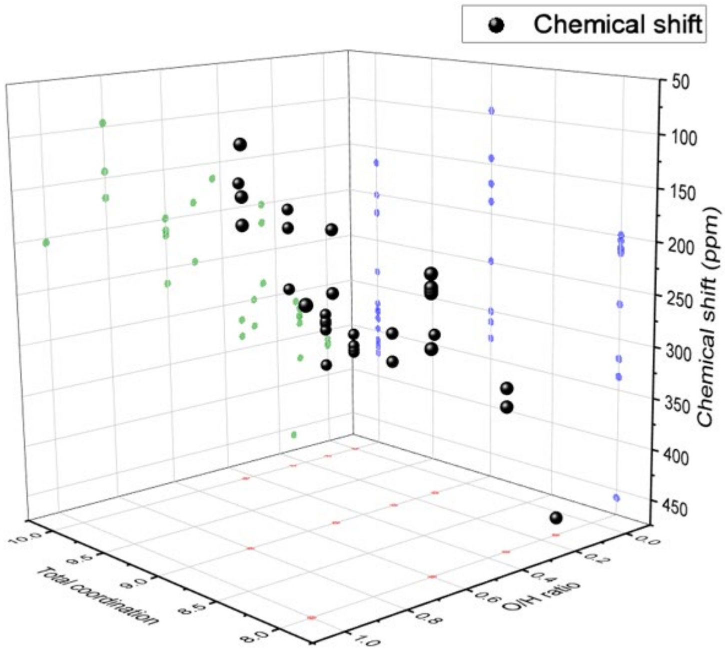

Figure S14: 3-D plot showing the factors which affect the ^{89}Y chemical shift values for a specific composition (*x* in $YO_xH_{(3-2x)}$ for disordered anion lattices or SQS). The 2D projection identified by green dots is a 2D plot correlating the ^{89}Y chemical shift and the O/H coordination ratio in the first coordination shell. This clearly shows that the yttrium nuclei become more shielded with higher O/H coordination ratios. The blue dots on the side show a 2D plot between total coordination number and the yttrium chemical shift, which is highly scattered not showing any correlation. Therefore, the yttrium chemical shift values are predominantly determined by the O/H coordination.

S15: ^{17}O deconvoluted spectrum of Yttrium oxyhydride thin film

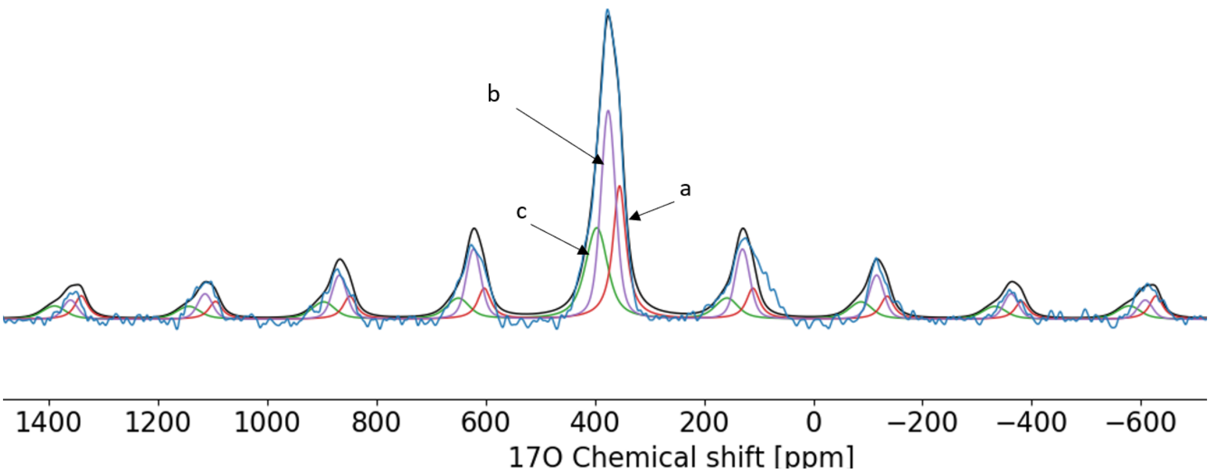

Figure S15: Full deconvolution of the ^{17}O MAS spectrum measured at 400 MHz and 19 kHz spinning frequency. The ratio of the satellite transition to the central transition (integral) can give a good estimate of the quadrupolar parameters (as listed in Table-3 in the main text).

Table S16: Table S1 Calculated quadrupolar parameters (C_Q and η) for ^2H and ^{17}O in $YO_xH_{(3-2x)}$ SQS structures with x=0.25 and x=1 (edge length= 10.65Å).

Nucleus	x in ($YO_xH_{(3-2x)}$)	Calculated. <|C_Q|> (MHz) for $YO_X H_{(3-2X)}$	Calculated <η> for $YO_X H_{(3-2X)}$
^2H	1.00	0.014 (H^-)	0.610 (H^-)
	0.25	0.013 (H^-)	0.542 (H^-)
	0.25	0.177 (H_2)	0.081 (H_2)
^{17}O	0.25	0.679 (O^{2-})	0.616 (O^{2-})
	0.25	7.143 (OH^-)	0.028 (OH^-)
	1.00	0.835 (O^{2-})	0.614 (O^{2-})
	1.00	6.174 (OH^-)	0.152 (OH^-)

S17: ^{1}H calculated chemical shift after O-H incorporation into the model SQS $YO_xH_{(3-2x)}$ (edge length= 10.65Å) and 1H regression plot.

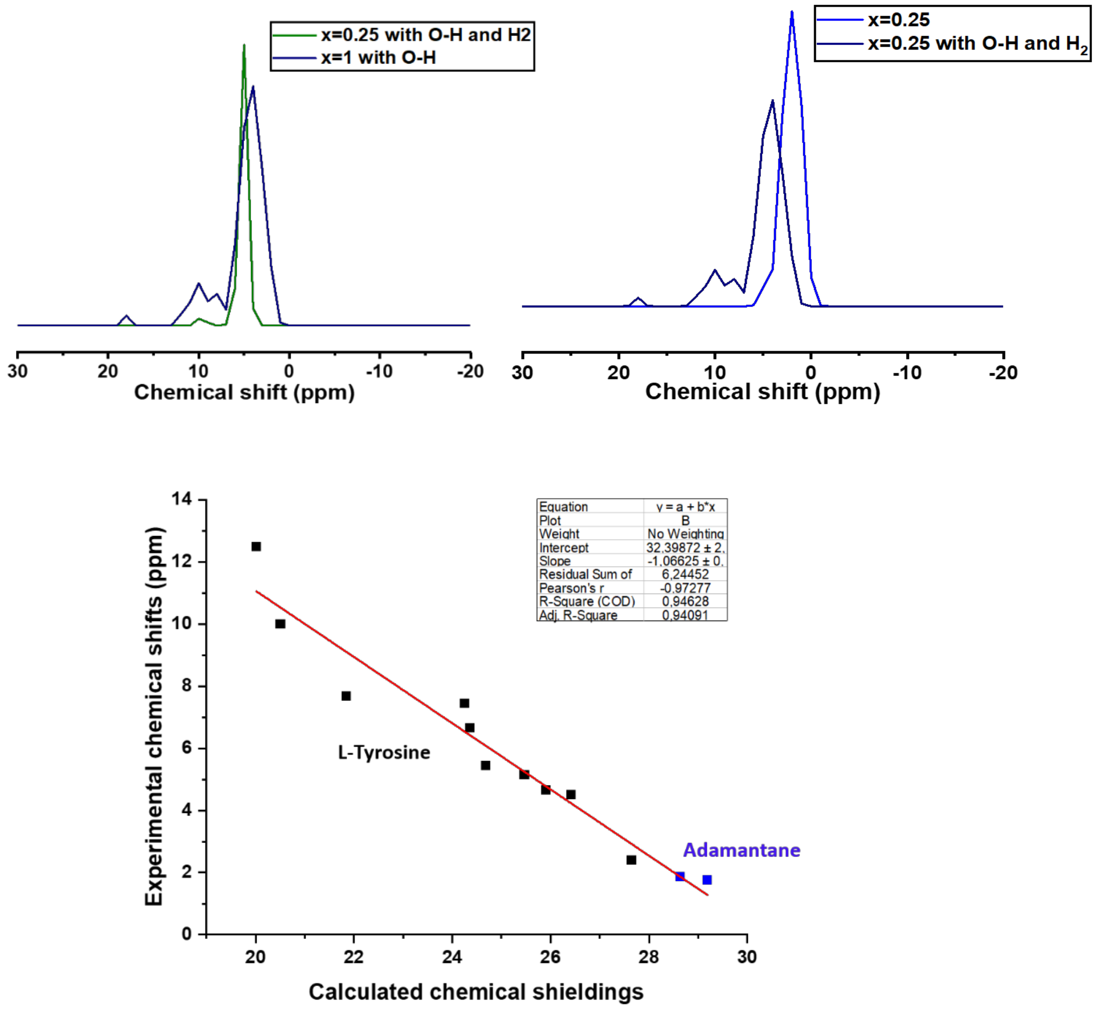

Figure S17: (top, left) Overlay of calculated 1H spectra of model O-H group incorporated yttrium oxyhydride SQS structure with x=0.25 and x=1, (top, right) overlay of 1H calculated spectra of yttrium oxyhydride SQS structures with and without O-H groups incorporated (bottom) regression plot to correlate calculated and experimental ^{1}H chemical shifts (for L-Tyrosine and Adamantane)

Model structures with O-H were constructed by incorporating O^{2-} and H^+ ions at a distance of 1.2 Å. These ions are inserted by replacing H^- ions as they are expected to be more labile than the O^{2-} ions. Seven O^{2-} and H^+ ions were inserted in both x=0.25 and x=1 compositions, using a randomly disordered anion sub-lattice. The H_2 formation (bond distance 0.75 Å) was uniquely observed for the x=0.25 (hydride rich) composition, whereas the x=1 lattice only formed O-H bonds. Therefore, attributing the narrow component in ^{1}H MAS spectrum to H_2, it can be assumed that H_2 is present only in the hydride rich domains. These are qualitative studies which indicate that such a process occurs upon O-H incorporation. Quantitative analysis, cannot be performed as the O-H might not be the only source of H_2 formation and it is not confirmed further, if there are other chemical species present in the narrow ^{1}H MAS peak.

S18: Matlab scripts used for simulated gaussian distribution for calculated H, Y and O chemical shifts

```
function [gauss, w1]= gaussian_distribtion1(mu, FWHM)
sigma1 = FWHM/(2*sqrt(2*log(2)));
w1 = -2000:2000;
p1 = -.5 * ((w1 - mu)/sigma1) .^ 2;
p2 = (sigma1 * sqrt(2*pi));
gauss = exp(p1) ./ p2;
end

Data= importdata('1H_YOOH_100.txt');
a=Data(:,1);
sz = size(a);
[gauss1, w1]= gaussian_distribtion1(9.6404,1);
for i = 2:1:sz
    [gauss, w1]=gaussian_distribtion1([a(i,1)],1);
    gauss1= gauss1+gauss;
end
Data= importdata('1H_YOOH_25.txt');
b=Data(:,1);
sz = size(b);
[gauss2, w2]= gaussian_distribtion1(10.9998,1);
for i = 2:1:sz
    [gauss, w2]=gaussian_distribtion1([b(i,1)],1);
    gauss2= gauss2+gauss;
end

plot(w1, gauss1,'r');
hold on
plot(w2, gauss2, 'b');
```

S19: Solid State NMR experimental details:

^{1}H, ^{2}H and ^{89}Y experiments: ^{1}H single pulse excitation (SPE), ^{1}H spin echo spectra and inversion recovery experiments were acquired using a 90° pulse at an RF field strength of 140 kHz, a 20 kHz MAS frequency, a recycle delay of 10s and 16 transients. ^{1}H-^{1}H Exchange Spectroscopy was performed to study spin diffusion between ^{1}H sites under the above conditions with mixing time of 10ms. For the ^{89}Y CP-MAS experiments, 20 kHz MAS was employed with an ^{89}Y RF field strength of 40 kHz and a ^{1}H field strength of 60 kHz. Ramped cross-polarisation experiments were used at an optimised contact time of 8ms and 32000 transients for the YHxOy samples. SPINAL-64 proton decoupling at an RF field strength of 85 kHz was employed during acquisition.

2D Inverse detected Lee Goldberg Cross Polarisation[1] was performed to suppress ^{1}H spin diffusion and selectively probe interactions between yttrium and hydrogen employing a 38 kHz ^{1}H RF field for CP. The effective ^{1}H RF field (B_{1H} /sin(54.73)) obtained was 46.54kHz with an offset of 26.87 kHz ($B_{1H}/\sqrt{2}$), for the second polarisation transfer to establish the Lee Goldberg decoupling condition. The spinning speed used for the experiment was 10 kHz and thus the RF field on the ^{89}Y channel was set to 36.87kHz.

For static ^{1}H measurements, a proton RF field strength of 50 kHz strength was used to perform single pulse measurements, with 5.2 us 90 °pulse length and recycle delay of 5s.

^{2}H SPE and VT measurements using a hard pulse of 70kHz RF field strength in order to excite all the satellite transitions. A small tip angle (π/3; pulse length 2.3us) and a recycle delay of 4s was utilised for the measurements. SPINAL-64 ^{1}H decoupling with RF field of 85kHz was used during acquisition. 1k transients were acquired for each measurements to obtain good signal to noise ratio.

^{17}O and ^{17}O-^{1}H correlation experiments: Single pulse experiments ^{17}O spectra were acquired with a hard pulse of RF field strength 68kHz, using a small tip angle of π/6 (1.2us), CT selective pulses had an RF field strength of 2kHz (31.25us, π/8). SPINAL-64 ^{1}H decoupling with RF field of 85kHz was used during acquisition. The recycle delay used for these measurements were 4s and 2k transients were taken to obtain good signal to noise ratio.

For the PRESTO recoupling experiments, the ^{1}H recoupling pulses were rotor synchronized and the symmetry used for these experiments were $R18_2^5$ with a MAS rate of 15kHz and ^{1}H decoupling power of 85 kHz. The recoupling power used on the ^{1}H channel was 4.45[2,3] times the MAS which was 66.75 kHz. The ^{17}O RF field used for the experiments was 12kHz central transition selective pulse with a solid 90 degree pulse width of 6.9us. The recycle delay used for these measurements were 4s and 80k transients were acquired.

Table S20: Comparing the large and small $YH_xO_{(3-2x)}$ cell NMR parameters

**Supercell edge length (Å)**	**x in $YO_xH_{(3-2x)}$**	**Quadrupolar coupling constant <|Cq|> (MHz)**		**Assymetry parameter <|η|>**		**^{17}O chemical shift range (ppm)**	**^{1}H chemical shift range (ppm)**
		^{17}O	^{2}H	^{17}O	^{2}H		
10.65<br>(Small cell)	0.25<br>1.00	0.64<br>0.79	0.012<br>0.016	0.69<br>0.65	0.50<br>0.63	300-450<br>350-500	1.7-5.1<br>4.7-5.8
21<br>(Large cell)	0.25<br>1.00	0.68<br>0.83	0.014<br>0.013	0.61<br>0.61	0.542<br>0.610	315-470<br>358-525	2.1-5.6<br>4.9-6.3

DFT calculations for small and large cells for both x=0.25 and x=1 give consistent NMR parameters.

S21: Deconvolution of the ^{17}O MAS spectrum (15kHz spinning frequency) with the O-H group

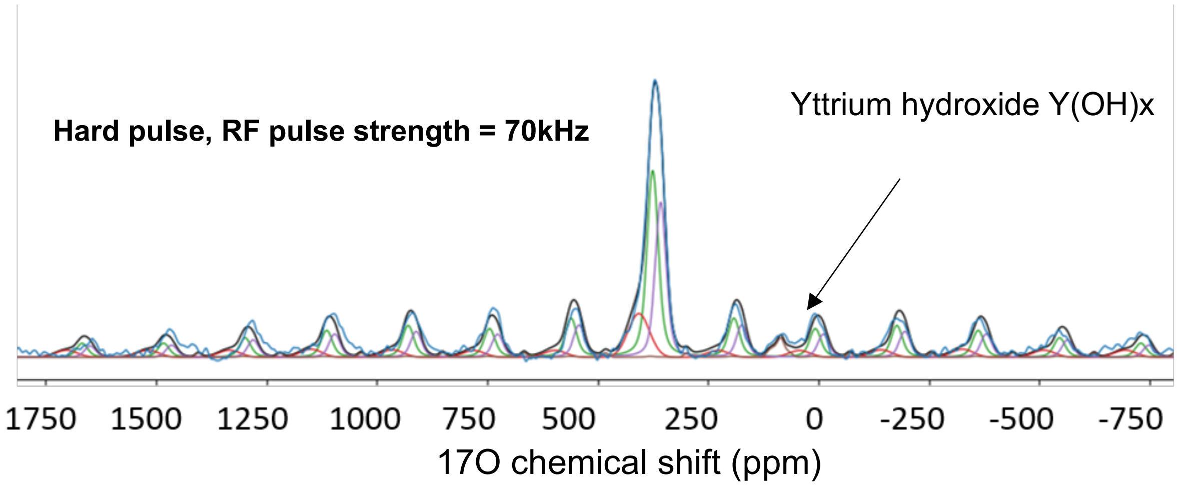

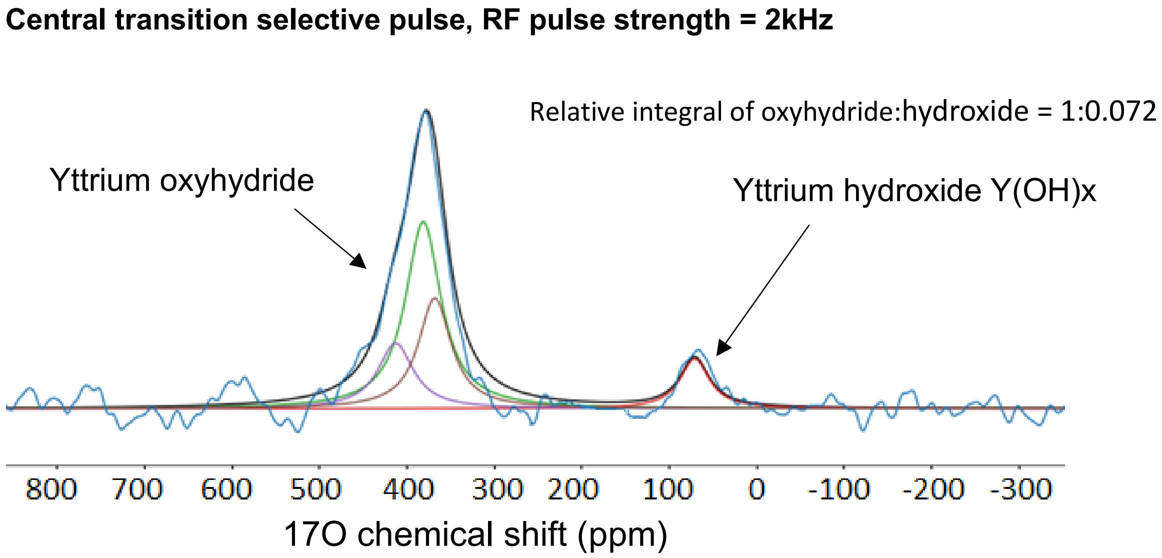

Figure S21: (top) ^{17}O spectrum of yttrium oxyhydride thin film (deposited at 0.5Pa H_2 dep. pressure) using a hard pulse of 70 kHz RF field strength, (bottom)) ^{17}O spectrum of yttrium oxyhydride thin film (deposited at 0.5Pa H_2 dep. pressure) using a central transition selective pulse of 2kHz RF field strength. The CT selective spectrum is obtained by adding the central transition selective spectra of the oxyhydride and the hydroxide peak (on the right). In both cases the RF field used was 2kHz. This technique was useful to remove the contribution from satellite transitions and allow the quantitative evaluation of the peak intensities.

Assuming a 3:2 ratio of hydrogen-rich (x=0.25) and hydrogen poor (x=1) domains, the total number of O (in $YO_xH_{(3-2x)}$) will be 3/5 * (0.25) + 2/5 * (1)= 0.55. Therefore, the absolute number of O-H will be 7.2% of 0.55 = 0.0396. Hence, in the ^1H spectrum the absolute number of O-H will also be 0.0396. The number of H in the ^1H spectrum is (3-2x), hence for x=0.25 it is 2.5 and for x=1, it is 1. As a result the absolute no. of H in the ^1H spectrum is : 3/5 * (2.5) + 2/5 * (1) = 1.9. Comparing this to the number of hydroxide (OH) protons, we see their occurrence amounts to (0.0396/1.9) * 100 = 2.1%. Therefore it will not be possible to distinguish the OH groups in he proton spectra.

S22: Comparing simulated and experimental ^{17}O spectrum for SQS $YO_xH_{(3-2x)}$

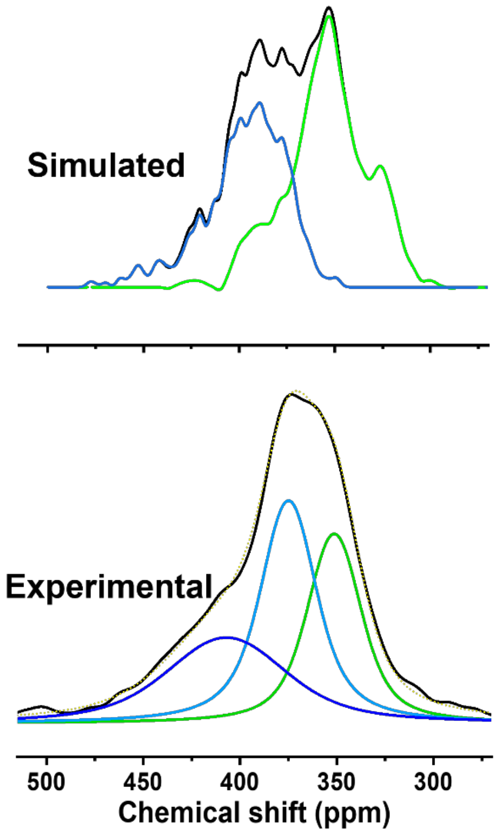

Figure S22: Comparing 17O experimental spectrum of yttrium oxyhydride thin film with the calculated ^{17}O spectrum of model yttrium oxyhydride large cells with anion disordered sub-lattice constructed by taking the integral ratio of the hydride rich (with x=0.25) and hydride poor (with x=1.00) domains into consideration (in the ratio of 3:2 as obtained from ^{1}H NMR studies).

The structures used for the simulated plot were large cells with an edge length of 20 Å (256 Y, O and H atoms for x=1.00 and 256 Y, 64 O and 640 H atoms for x=0.25). Thus based on the total no. of oxygen atoms for each composition and also the integral ratio obtained from ^{1}H NMR studies (0.7:0.2), the domain size (relatively) of the hydride rich (x=0.25) and hydride poor(x=1.00) domains is obtained to be nearly 3:2. The simulated spectra are constructed from the calculated shifts using the Matlab script in S-18.

S23: [1]H Quantitative studies of Yttrium oxyhydride thin films (empirical formula determination)

Table S23: Comparing the weight and the integral value of Glycine and Yttrium oxyhydride powder deposited at 0.5 Pa H_2 dep. pressure

Sample	Weight (mg)	Integra
Glycine	7.9	7.6188
YHO-0.5	12.3	4.0269

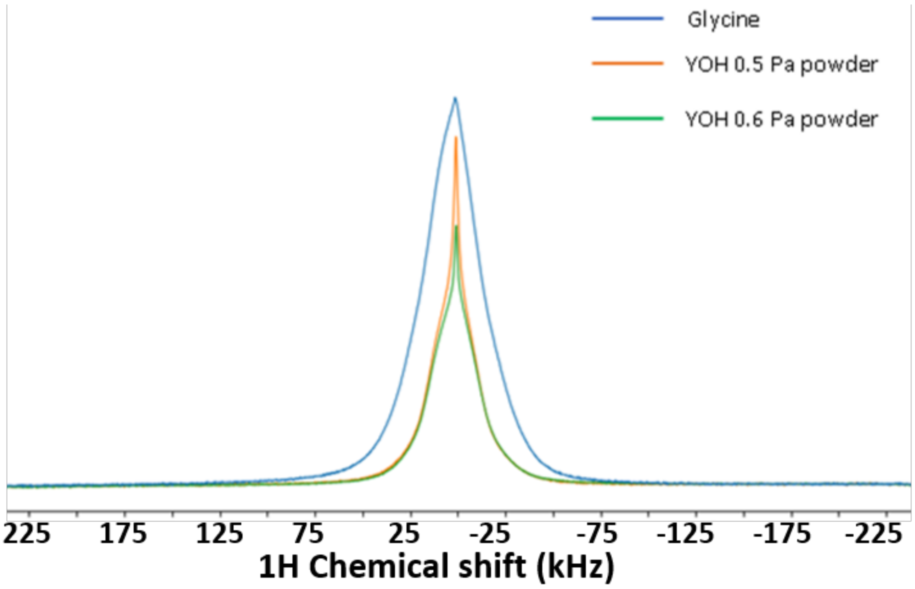

Figure S23: Comparing the static ^{1}H spectra of Glycine, Yttrium oxyhydride powder deposited at 0.5 and 0.6 Pa H2 dep. pressure.

7.9 mg of glycine corresponds to $\frac{7.9*5}{75.07} = 0.526$ mmol H in the sample, giving an integrated intensity of 7.62. Here we used that the molecular weight of glycine is 75.07

12.3 mg of the oxyhydride YHO-0.5 gives a relative intensity of 4.03. Considering the fact that 10% of the signal intensity in the ^{1}H spectrum is due to trapped H_2 (narrow line), the intensity attributed to the YHO fractions is 0.9*4.03=3.62 which, comparing to the intensity obtained for glycine, corresponds to 0.25 mmol H in the YHO fractions. As the contribution of the trapped H_2 to the mass of the sample is less than ~2% we can calculate the average composition of the YHO fractions, using the atomic masses of yttrium, oxygen and H as respectively 88.905, 15.999 and 1, to be:

$$\frac{12.05 * (3 - 2x)}{88.905 + 15.999x + (3 - 2x)} = 0.25\, mmol\, H$$

Solving for x, we find a value of x=0.48.

S24: Extraction of ^{89}Y spectra of hydride rich (x=0.25) and hydride poor (x=1) domains

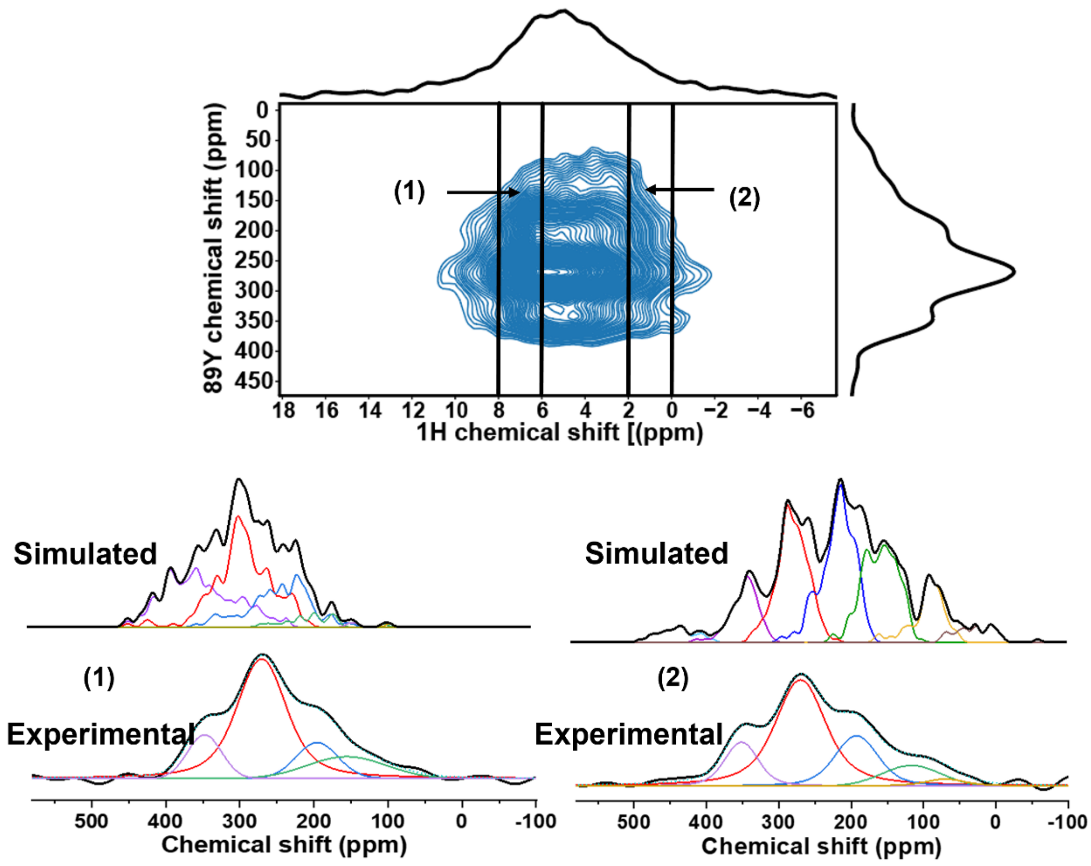

Figure S24: 2D ^{89}Y-^{1}H HETCOR spectra and partial sum projections giving ^{89}Y spectra by summing over a limited ^{1}H chemical shift range (marked as 1 and 2 in the 2-D HetCor spectrum). The ^{1}H regions that were selected are 0-2 ppm for the hydride poor domains giving the ^{89}Y projection on the lower right and 6-8 ppm for the hydride rich domains giving the ^{89}Y projection on the lower left. The simulated spectra in the middle are constructed from the calculated shifts using the Matlab script in S18.

S25: Calculated total energies for various compositions of yttrium and lanthanum oxyhydrides (SQS and random models)

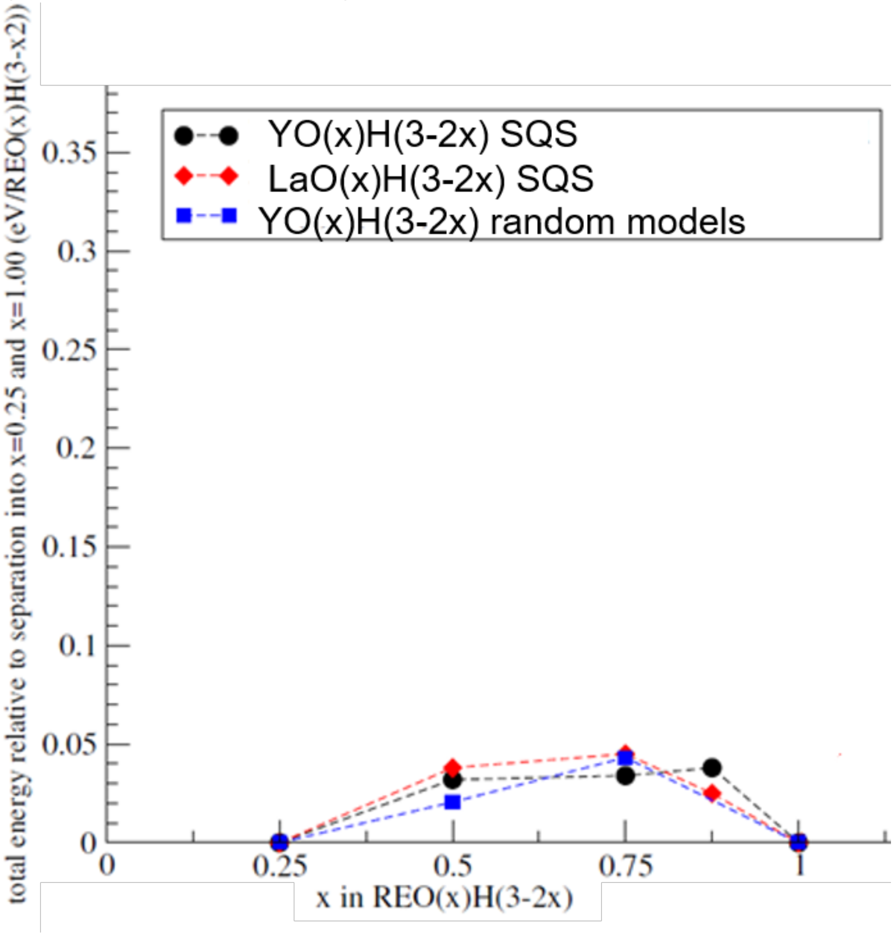

Figure S25: Calculated total energies relative to separation into x=0.25 and x=1.00 for various compositions of yttrium and lanthanum oxyhydrides (SQS and random models)

The process of calculating the above total energies is as follows:

x = alpha * x1 + (1 - alpha) * x2

Where x is the composition restricted to values < 1. Subsequently x is split into x1 and x2 where x1=0.25 and x2=1.00 and alpha is the fraction of x1 and (1-alpha) is the fraction of x2. Solve for alpha and then get an energy difference between a single composition or a two phase system:

e(x) - alpha * e(x1) - (1 - alpha) * e(x2)

This shows that for each of these compositions, a phase segregation into a hydride rich (x=0.25) and an hydride poor (x=1.00) is energetically favourable over a single composition.

S26: <u>DFT + U chemical shift calculations</u>

DFT +U methods are applied on the 4f orbital of Y to correct for the overestimated hybridization of the 2p O and the unoccupied 4f Y orbital, resulting in an increase in its covalent character. This could prevent an accurate calculation of the NMR parameters. The technique used to overcome this issue is to employ an artificial potential (U) on the Y 4f which effectively raises its energy, thus reducing the mixing of the orbitals. The value of U is taken to be 2.8 eV[4,5]. The reference compound used is Y_2O_3 and $YAlO_3$. However, the observed changes in chemical shift values for the Yttrium oxyhydride systems are negligible and do not influence the chemical shift trends.

Table S26: The table lists the applied potential energy (U), orbital with U, calculated shieldings with and without U for yttrium oxide and yttrium alluminium oxide.

Structure	Nucleus	U (potential energy) (eV)	Orbital with U	-Shielding with U	-Shielding without U
Y2O3	89Y	2.8	4f (Y)	-2419.51, -2373.72	-2421.45, -2376.50
Y2O3	17O	2.8	4f (Y)	150.53	156.21
YAlO3	89Y	2.8	4f(Y)	-2427.85	-2423.01
YAlO3	17O	2.8	4f(Y)	-59.77, -87.86	-61.82, -90.51

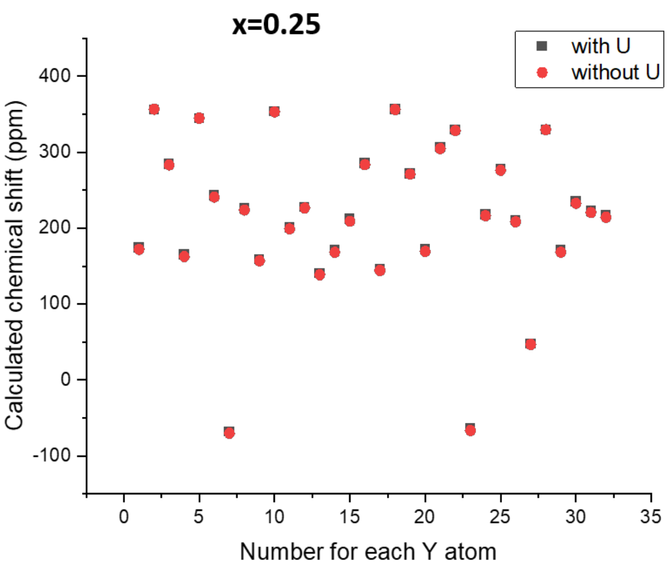

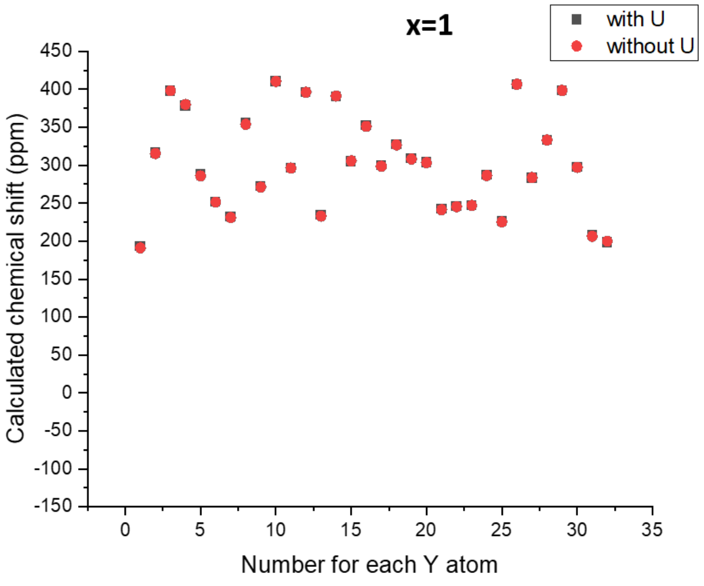

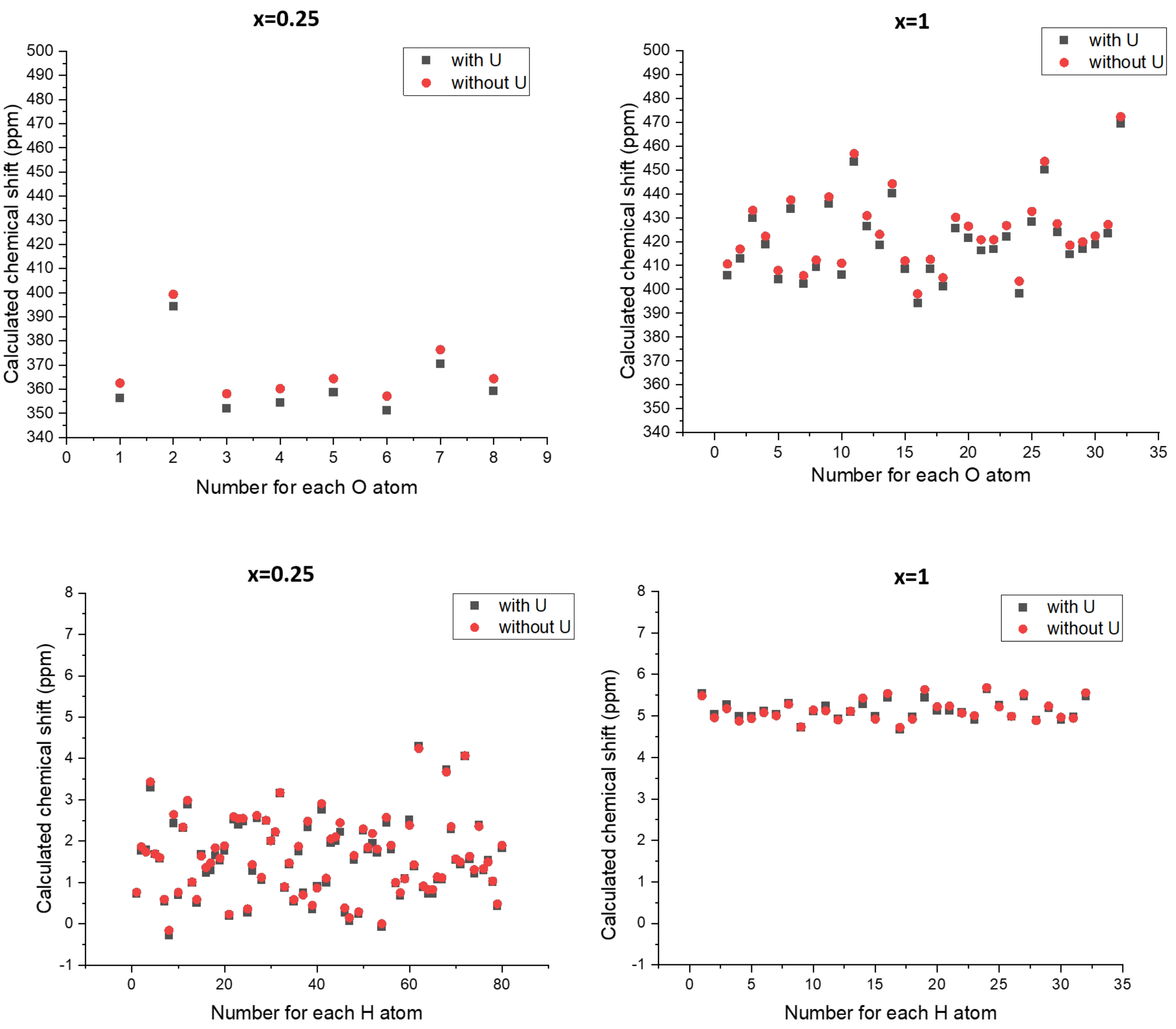

Figure S26: Comparing calculated 89Y (top), 17O (middle) and 1H (bottom) chemical shifts for x=0.25 and x=1.00 Yttrium oxyhydride SQS structures with and without applying U. ^{89}Y chemical shifts change by 0.05% on average, ^{17}O chemical shifts change by 2.1% on average and ^{1}H chemical shifts change by 1.35% on average.

REFERENCES:

(1) Ladizhansky, V.; Vega, S. Polarization Transfer Dynamics in Lee-Goldburg Cross Polarization Nuclear Magnetic Resonance Experiments on Rotating Solids. *J. Chem. Phys.* **2000**, *112* (16), 7158–7168. https://doi.org/10.1063/1.481281.

(2) Nagashima, H.; Trébosc, J.; Trébosc, J.; Kon, Y.; Sato, K.; Lafon, O.; Lafon, O.; Amoureux, J. P.; Amoureux, J. P.; Amoureux, J. P. Observation of Low-ΓQuadrupolar Nuclei by Surface-Enhanced NMR Spectroscopy. *J. Am. Chem. Soc.* **2020**, *142* (24), 10659–10672. https://doi.org/10.1021/jacs.9b13838.

(3) Gómez, J. S.; Rankin, A. G. M.; Trébosc, J.; Pourpoint, F.; Tsutsumi, Y.; Nagashima, H.; Lafon, O.; Amoureux, J. P. Improved NMR Transfer of Magnetization from Protons to Half-Integer Spin Quadrupolar Nuclei at Moderate and High Magic-Angle Spinning Frequencies. *Magn. Reson.* **2021**, *2* (1), 447–464. https://doi.org/10.5194/mr-2-447-2021.

(4) Jaworski, A.; Charpentier, T.; Stevensson, B.; Edén, M. Scandium and Yttrium Environments in Aluminosilicate Glasses Unveiled by 45Sc/89Y NMR Spectroscopy and DFT Calculations: What Structural Factors Dictate the Chemical Shifts? *J. Phys. Chem. C* **2017**, *121* (34), 18815–18829. https://doi.org/10.1021/acs.jpcc.7b05471.

(5) Shih, B. C.; Yates, J. R. Gauge-Including Projector Augmented-Wave NMR Chemical Shift Calculations with DFT+ U. *Phys. Rev. B* **2017**, *96* (4), 1–10. https://doi.org/10.1103/PhysRevB.96.045142.
